# AI‐Augmented Iterative Screening of Libraries Against RNA Targets (AISLAR) Boosts Discovery of SAR‐Tractable RNA Binders and Rational Analog Design

**DOI:** 10.1002/smsc.202600007

**Published:** 2026-04-17

**Authors:** Haruhiko Hattori, Maina Otsu, Koji Imai, Mebuki Narahara, Jiro Kondo, Amiu Shino, Ella Czarina Morishita

**Affiliations:** ^1^ Veritas In Silico Inc. Tokyo Japan; ^2^ Department of Materials and Life Sciences Faculty of Science and Technology Sophia University Tokyo Japan

**Keywords:** drug discovery, iterative screening, machine learning, medicinal chemistry, RNA structures

## Abstract

Small molecules that target RNA are emerging as a powerful therapeutic modality, although deriving structure–activity relationships (SARs) remains a major challenge. Here, we present AI‐augmented Iterative Screening of Libraries Against RNA targets (AISLAR), a machine learning‐driven strategy that accelerates the discovery of SAR‐tractable RNA binders and enables rational analog design. We screened diverse, drug‐like chemical libraries against two RNA motifs derived from human p53 mRNA and applied AISLAR within the open‐source KNIME platform. The application of AISLAR yielded chemotypes suitable for SAR development. Biophysical assays confirmed direct binding of representative compounds to one RNA motif. Guided by early SAR trends, we developed a pharmacophore hypothesis and designed an analog that retained binding with lower predicted cardiac channel liability. Docking simulations using the crystal structure of the RNA motif revealed a plausible binding mode for the validated hit compound. While further validation across diverse RNA targets and compound libraries will be required, these results demonstrate how AISLAR can be used as a workflow linking RNA‐targeted small molecule screening with rational analog design.

## Graphical abstract

AI‐augmented iterative screening of libraries against RNA targets (AISLAR) combines fluorescence resonance energy transfer screening, machine learning, and validation by dose response and isothermal titration calorimetry to discover RNA binders. Applied to p53 mRNA motifs, AISLAR identified chemotypes with tractable structure–activity relationships, guiding analog design through docking‐informed pharmacophore models.

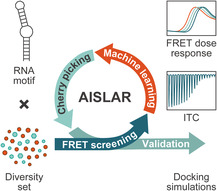

## 1 Introduction

RNA has emerged as a compelling target for small molecule therapies. However, despite substantial efforts, only a few successful examples have been reported, most identified through phenotypic screening campaigns [1, 2, 3]. A major limitation of phenotypic screening is that it rarely provides tractable structure–activity relationships (SARs), thereby limiting rational analog design. In contrast, protein‐targeted drug discovery has seen significant advances over recent decades. Target‐based approaches in this field have proven effective for generating SAR‐tractable hit series, facilitating the rational design of compounds with optimized pharmacological and toxicological profiles [4]. These successes were largely supported by cell‐free assay systems and structure‐based drug design (SBDD). Meanwhile, similar enabling technologies for RNA‐targeted small molecule drug discovery remain scarce.

To address this gap, we previously developed a target‐based workflow for RNA ligand discovery that integrated high‐throughput screening (HTS), hit validation, and lead generation using SBDD [5]. The next challenge is to increase the likelihood of obtaining highly SAR‐tractable hit series from HTS campaigns. Two potential solutions are focused library screening and iterative screening. Focused libraries remain limited by incomplete knowledge about druggable RNA motifs and RNA‐binding chemotypes, although several pioneering studies have begun to define chemical features enriched in such small molecules [6, 7, 8, 9, 10, 11, 12, 13, 14, 15, 16, 17].

On the other hand, iterative screening [18, 19, 20, 21, 22, 23, 24, 25]—in which compounds are tested in batches and each subsequent batch is selected using chemoinformatic analysis of prior results—could be applied to any type of target, including RNAs with unknown 3D structures. Moreover, this process may allow early estimation of the total number of active compounds in a library and help prioritize RNA motifs with higher hit rates when screening multiple targets in parallel. Although not focused on RNA‐targeted small molecules, the study by Dreiman et al. [23] provides a robust workflow for machine learning (ML)‐assisted iterative screening, covering subset selection, compound representation, algorithm optimization, and iteration design. Advances in computational chemistry tools now enable medicinal chemists to integrate ML into iterative screening workflows. In particular, low‐code platforms such as KNIME [26], widely adopted in the chemistry community, offer accessible modules for implementing iterative screening simulations.

**FIGURE 1:**
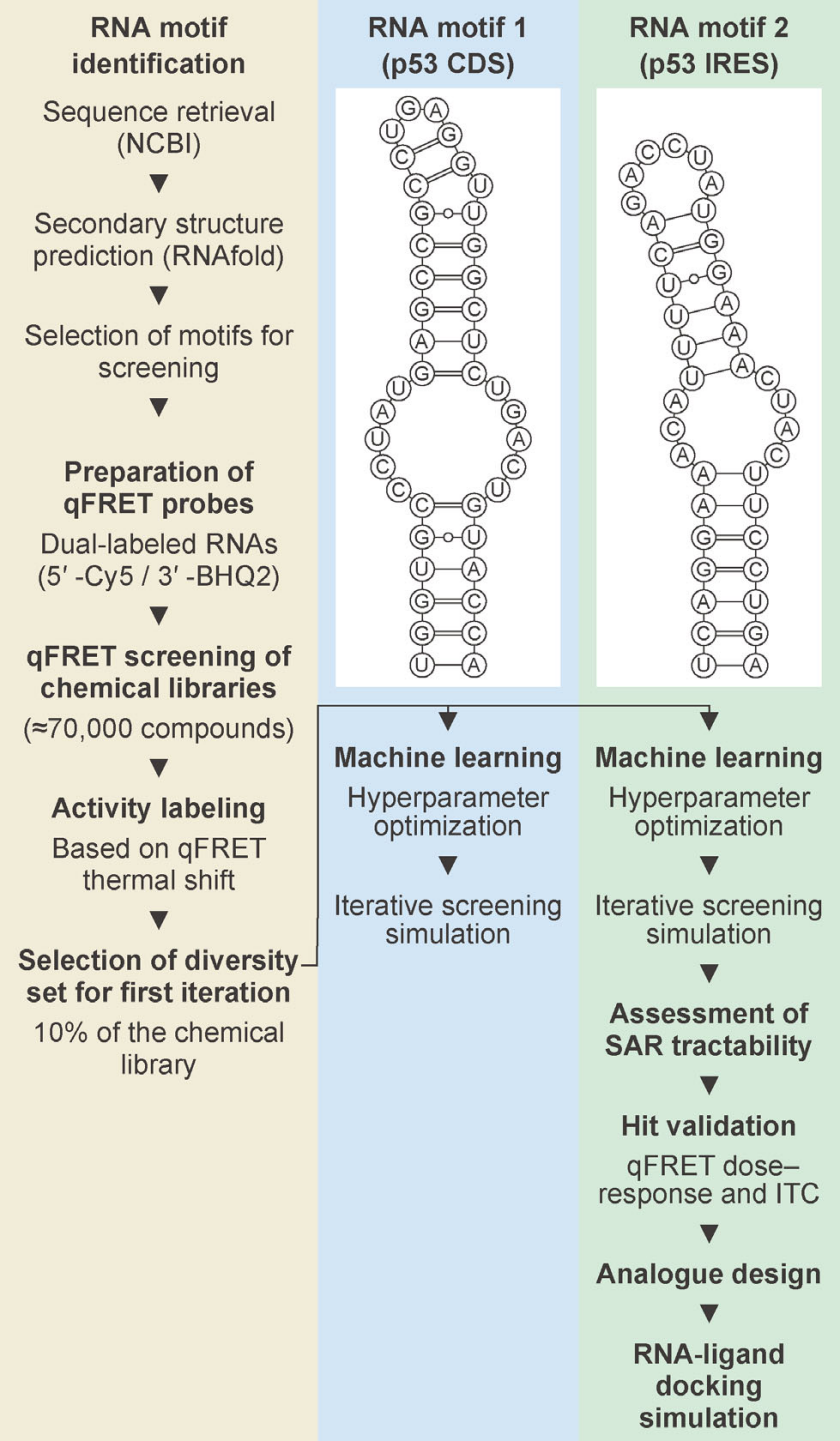
Overview of the workflow employed in this study. Three distinct chemical libraries were screened against two p53 RNA motifs, 1 and 2 (secondary structures shown). Machine learning algorithms were independently trained and optimized using the first iteration dataset for RNA motifs 1 and 2, and iterative screening simulations were conducted. The optimized model was then applied to RNA motif 2, with hit validation carried out using quantitative fluorescence resonance energy transfer (qFRET) dose–response assays and isothermal titration calorimetry (ITC). Subsequent steps included SAR tractability assessment, analog design, and docking simulations.

In this study, we present the first application of an iterative screening approach augmented by ML in RNA‐targeted small‐molecule drug discovery, termed AISLAR. We demonstrate how AISLAR can enhance HTS productivity relative to nonprioritized screening and enable the identification of SAR‐tractable hit compounds suitable for rational analog design. Our workflow combines computational and biophysical techniques to support iterative screening and hit validation against two RNA motifs derived from human p53 mRNA (RNA motifs 1 and 2) (Figures 1 and S1, Supporting Information).

p53 is a central tumor suppressor whose expression is tightly regulated at multiple levels, including translation [27, 28]. The 5′ untranslated region of p53 mRNA contains conserved secondary structure elements that participate in translational control and contribute to the region's established internal ribosome entry site (IRES) activity [29, 30, 31]. Among these elements, the U187–A225 stem‐loop used in this study (RNA motif 2) lies within the IRES‐active region and serves as a binding platform for multiple translational regulators, including HDM2, HDMX, PTB, SFPQ, and hnRNP C1/C2, which modulate p53 mRNA conformation and influence translational efficiency under stress conditions [32, 33, 34, 35, 36, 37]. Because dysregulated p53 translation is implicated in oncogenic transformation and stress responses [38], small molecules that bind to these RNA structures could, in principle, modulate p53 levels independently of canonical DNA damage pathways, providing a distinct entry point for therapeutic intervention. By stabilizing or perturbing structured elements within the p53 IRES, small‐molecule binders of RNA motif 2 could alter the efficiency of cap‐independent p53 translation under stress conditions, thereby modulating p53 protein levels and downstream tumor‐suppressive responses.

To assess selectivity within the screening workflow, we also included a structurally distinct stem‐loop (RNA motif 1) that does not overlap with known p53 regulatory regions. This enabled us to distinguish motif‐preferential binders from general RNA binders and to examine how iterative screening enriches for compounds that preferentially interact with biologically meaningful motifs.

We began by assembling a chemically diverse screening library consisting of the Food and Drug Administration (FDA)‐Approved Drug Library from MedChemExpress, and the Discovery Diversity Set (DDS) and Representative Diversity set (RDS) from Enamine. We examined the behavior of the workflow by performing simulation studies on the two RNA motifs independently. First, we screened the combined library against both RNA motifs using a fluorescence resonance energy transfer (FRET)‐based assay. We then independently trained ML algorithms on the first iteration dataset and performed iterative screening simulations on the HTS dataset for RNA motifs 1 and 2. Downstream analyses were intentionally focused on RNA motif 2, for which biological relevance, validation using orthogonal biophysical techniques, and tractable SAR were observed. Finally, we determined the X‐ray crystal structure of an RNA construct containing the internal loop of RNA motif 2 and performed docking simulations to visualize interactions with the validated hit compound, providing a testable hypothesis to support subsequent SBDD.

## 2 Results and Discussion

### 2.1 Screening of Chemical Libraries

We screened the combined chemical library against RNA motifs 1 and 2 using the previously described quantitative fluorescence resonance energy transfer (qFRET) assay [5, 39]. In this method, the RNA is labeled at the 5’ end with a fluorescence donor and at the 3’ end with a fluorescence acceptor. At low temperatures, the RNA is folded, allowing resonance energy transfer from donor to acceptor. As the temperature gradually increases, the RNA unfolds, increasing donor–acceptor distance and reducing energy transfer. This results in an increase in donor fluorescence, enabling measurement of the RNA melting temperature (*T*_m_). A positive change in *T*_m_ (Δ*T*_m_) indicates stabilization of the folded RNA motif. For the qFRET assays, we used 5’‐Cyanine5 (5′‐Cy5) and 3’‐Black Hole Quencher 2 (3′‐BHQ2) dual‐labeled RNA motifs. Compounds with Δ*T*_m_ greater than 1°C were classified as “active.” This cutoff was validated by binding studies of representative hits using orthogonal biophysical methods described below. The number of actives and hit rates for each RNA motif are summarized in Table 1.

**TABLE 1:** Active compounds and their ratios to valid data for the high‐throughput screening dataset on RNA motifs 1 and 2.

	Number of compounds	Ratio to valid data
Valid data	71 653	100%
Actives against RNA motif 1	216	0.30%
Actives against RNA motif 2	260	0.36%
Actives against both RNA motifs	153	0.21%

### 2.2 Iterative Screening Simulations

Next, we performed compound representation and iterative screening simulations using KNIME (version 5.4.0, available free of charge at https://www.knime.com). Each compound was represented using extended‐connectivity fingerprints (Morgan fingerprints, radius 2, 1024 bits) [40] and physicochemical descriptors (a total of 119). Both fingerprints and descriptors were calculated using the RDKit software package [40, 41], combined, and used to train ML models. We employed the Random Forest algorithm [42] because prior studies have demonstrated its methodological robustness for iterative screening of various public HTS datasets [23] and its utility in predicting small molecule binding to CAG repeats [43].

We began iterative screening using an initial 10% subset of the combined library selected using the RDKit Diversity Picker node, which applies MaxMin algorithm to select diverse compounds based on Tanimoto distance [44]. Hyperparameter tuning was independently performed on this first iteration dataset for RNA motifs 1 and 2 using the AutoML module of the H2O.ai platform (H2O‐3, version 3.46.0.1). The optimized hyperparameters were then used to construct classification models that predict the activity of the remaining compounds.

To determine an appropriate iteration size, we evaluated the enrichment factor (EF) as a function of the number of active compounds recovered within the top x% of predicted scores (Figure S2, Supporting Information). The total number of compounds and hits were defined based on the full library, including previously tested compounds. Selecting the top 5% of compounds yielded EFs of 5.5 for RNA motif 1 and 6.5 for RNA motif 2, which were considered sufficient to demonstrate the utility of the iterative screening approach. Accordingly, compounds were ranked by their predicted probability scores, and the top 5% were selected for the next iteration. After each iteration, the model was retrained using the updated dataset to refine compound selection.

**FIGURE 2:**
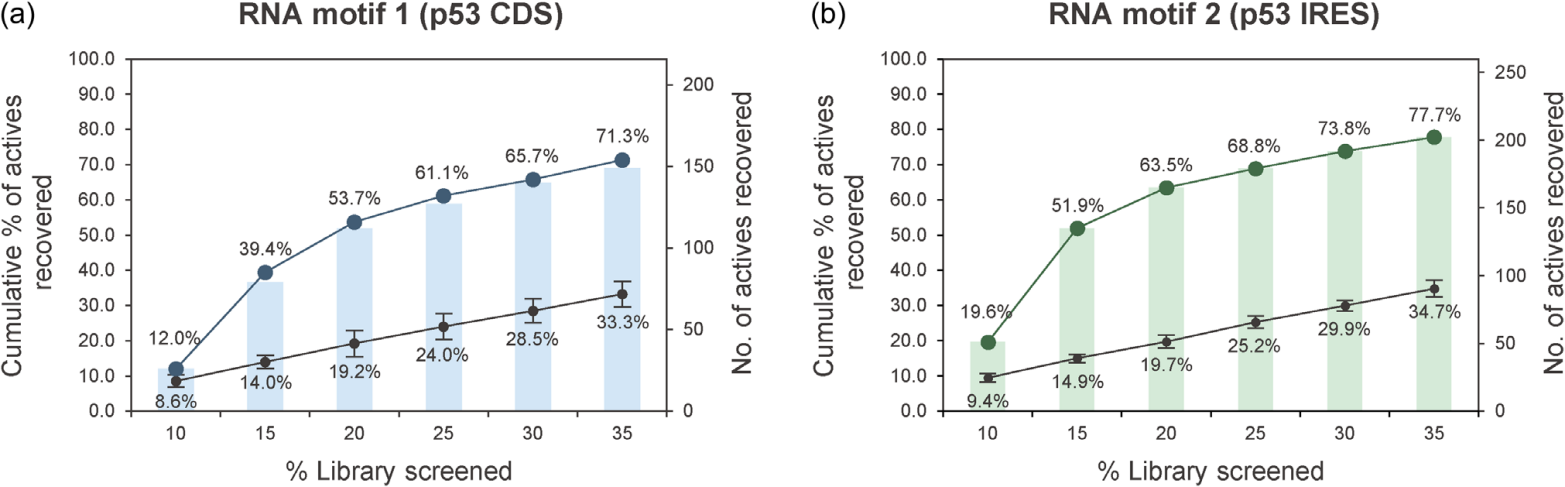
Cumulative percentages of actives recovered (left axis) and numbers of actives recovered (right axis) are shown for (a) RNA motif 1 (p53 CDS; blue) and (b) RNA motif 2 (p53 IRES; green) as increasing fractions of the combined chemical library are screened. Black curves indicate the expected recovery from random selection and represent the mean ± standard deviation of six independent random selections performed using different random seeds.

The recovery rates of active compounds are shown in Figure 2. For RNA motif 1, 71.3% of actives were recovered by screening only 35% of the library. Applying the same workflow to RNA motif 2 similarly improved the recovery efficiency. In contrast, random compound selection resulted in an approximately linear recovery of actives and consistently underperformed compared with AISLAR. These results demonstrate that AISLAR substantially enhances HTS productivity for the RNA motifs examined in this study. The overlap of active compounds recovered by iterative screening for RNA motifs 1 and 2 was comparable to those observed in the full HTS dataset (Figure S3, Supporting Information), suggesting that the workflow does not introduce additional selection bias.

### 2.3 Analysis of Actives Recovered by Iterative Screening

Given the promising iterative screening results, we next examined the recovered actives from a medicinal chemistry perspective. Specifically, we analyzed: (1) the Δ*T*_m_ values of recovered versus unrecovered actives, (2) their distribution in chemical space, and (3) the structures of representative active compounds.

Iterative screening was applied to RNA motifs 1 and 2 in parallel using the same computational workflow, resulting in comparable early enrichment behavior for both motifs. Based on these observations, subsequent analyses were focused on a single representative motif. RNA motif 2 was selected based on its established biological relevance, and experimental binding confirmation by isothermal titration calorimetry (ITC) was performed for this motif. Accordingly, subsequent analyses were restricted to RNA motif 2.

**FIGURE 3:**
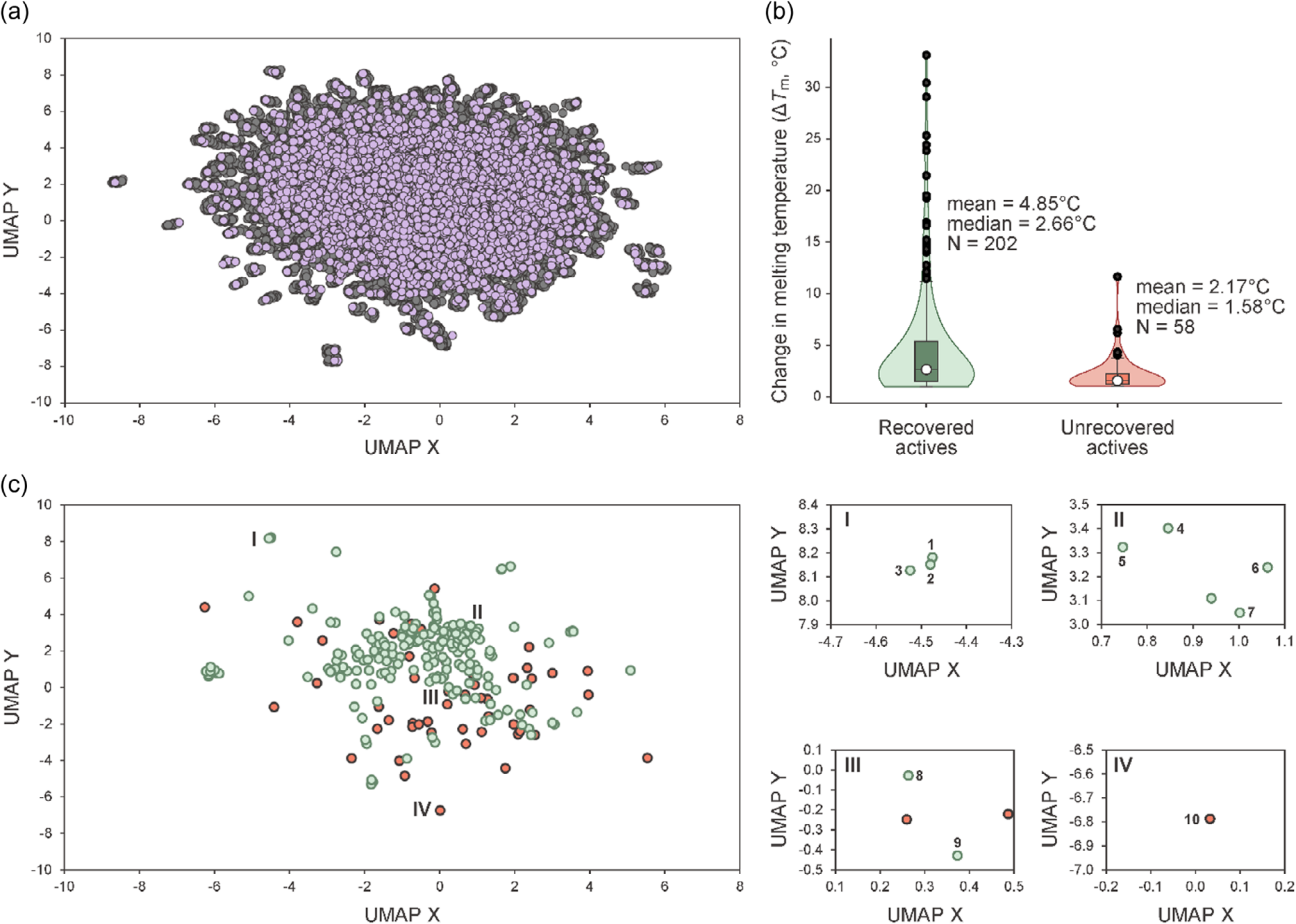
Visualization of chemical library selection and active compound recovery. (a) Uniform Manifold Approximation and Projection (UMAP) visualization of the combined chemical library, comprising three compound libraries. Each point represents a compound. Gray points indicate the full chemical space, while lavender points denote the initial compound set selected using the RDKit Diversity Picker. (b) Violin plot showing the distribution of change in melting temperature (Δ*T*_m_) values for recovered (green) and unrecovered (orange) actives. The white circle indicates the median. Mean, median, and sample size (N) values for each group are indicated. (c) UMAP projection of active compounds only, highlighting recovered (green points) and unrecovered (orange points) actives. The left panel shows the full distribution, while the right panels (I–IV) show magnified views of subregions where selected compounds discussed in the main text are located (numbered points).

To visualize the distribution of actives in chemical space, we applied Uniform Manifold Approximation and Projection (UMAP) [45]. As shown in Figure 3a, the initial compound set (lavender) covered a broad region of the chemical space defined by the full library (gray), confirming the effectiveness of the RDKit Diversity Picker in selecting a chemically diverse initial subset. As shown in Figure 3b, the recovered actives exhibited significantly higher mean and median Δ*T*_m_ values than the unrecovered actives, indicating preferential enrichment of stronger binders. We next visualized the distribution of active compounds using the same UMAP projection (Figure 3c). Most recovered actives (green; compounds **1**–**9**) were located near other active compounds within chemical space, indicating that they were recovered as distinct chemical clusters.

The structures and Δ*T*_m_ values of representative active compounds are shown in Table 2. Compounds **1**–**9** were recovered, while compound **10** was not. Notably, our method successfully identified clustered actives but failed to recover singletons. Compounds **1**–**3** and **8**–**9** share common structural features highlighted in blue and green colors, while compounds **4**–**7** were aminoglycoside antibiotics, which are well‐known RNA‐binding chemotypes. These findings indicate that our method preferentially recovers clustered active compounds over singletons, which is advantageous for SAR analysis and rational analog design.

**TABLE 2:** Selected Examples of Active Compounds.

ID	Structure	RNA motif 1 Δ*T*_m_,°C	RNA motif 2 Δ*T*_m_,°C
**1**	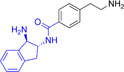	6.6	11.9
**2**	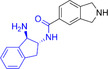	7.9	11.5
**3**	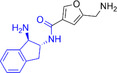	4.6	6.4
**4**	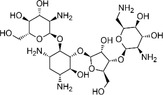	14.0	16.6
**5**	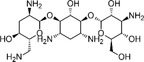	13.1	14.3
**6**	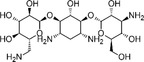	8.3	9.3
**7**	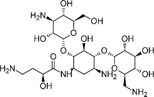	8.7	11.2
**8**	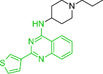	0.8	2.6
**9**	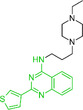	0.7	2.6
**10**	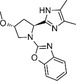	2.0	1.3

### 2.4 Biophysical Characterization of Active Compounds Recovered by Iterative Screening

**FIGURE 4:**
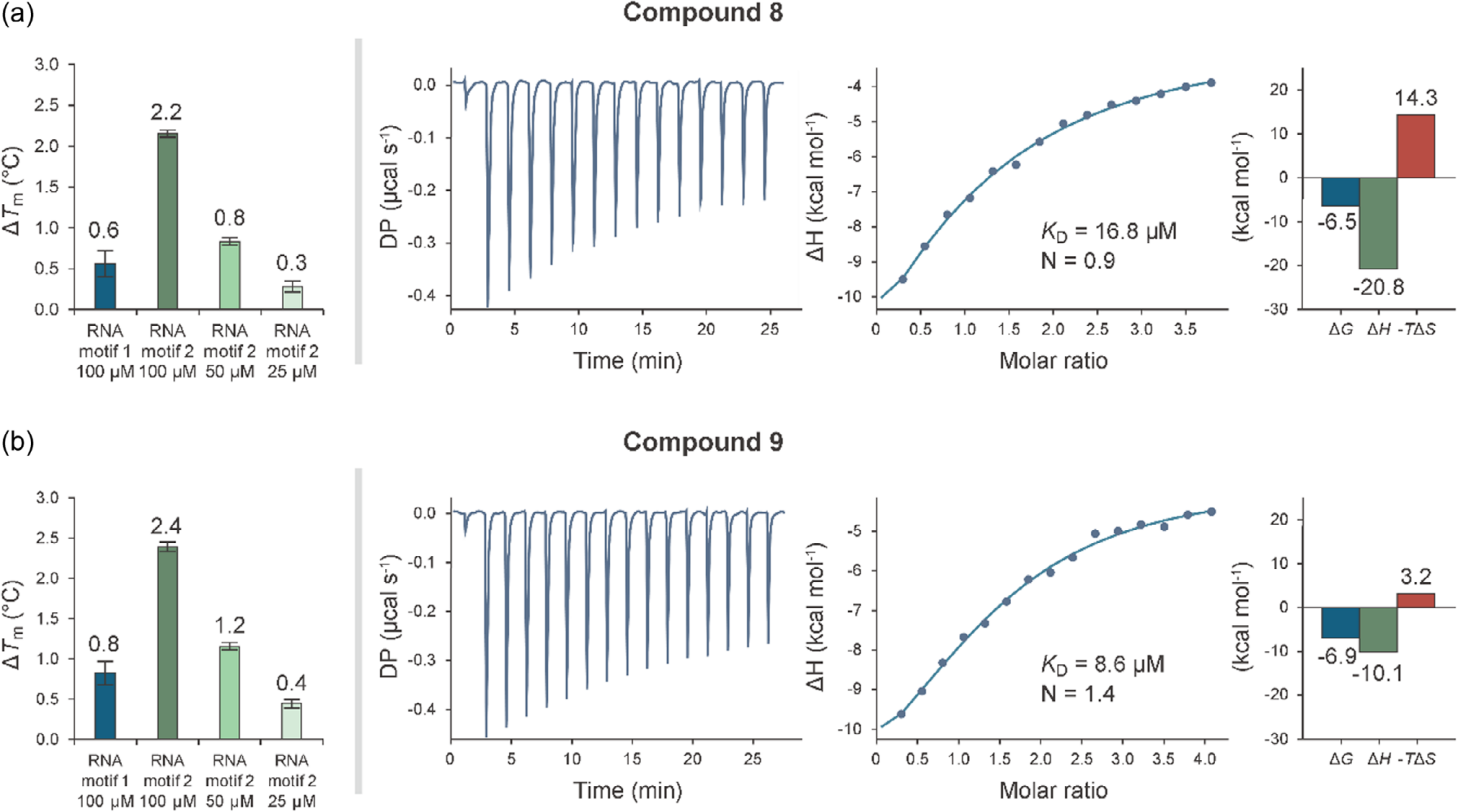
Biophysical characterization of compounds **8** and **9** binding to RNA motifs. (a) Data for compound **8**. First panel: qFRET dose–response analysis showing Δ*T*_m_ values for RNA motif 1 (blue) and RNA motif 2 (green). Second panel: ITC thermogram from titration of compound **8** into RNA motif 2. Third panel: Binding isotherm with fitted curve (single set of sites model), from which the dissociation constant (*K*_D_) and stoichiometry (N) were derived. Fourth panel: Thermodynamic parameters derived from ITC data: Δ*G* (blue), Δ*H* (green), and ‐*T*Δ*S* (red). (b) Data for compound **9**, organized as in (a).

To validate the interactions between RNA motif 2 and active compounds recovered by iterative screening, we performed qFRET dose–response assays and ITC (Figure 4) [46, 47]. Compounds **8** and **9** were selected for ITC experiments based on their selectivity for RNA motif 2 over RNA motif 1 in the qFRET assay and favorable dose–response profiles. These compounds bind to RNA motif 2 with dissociation constants (*K*_D_) of 16.8 and 8.6 μM, respectively. In contrast, no binding to RNA motif 2 was detected for compounds **1**–**3**. To investigate the reasons for this discrepancy, we conducted biolayer interferometry (BLI) measurements. Compounds **1**–**3** showed clear binding responses to RNA motifs 1 and 2 in BLI, although their sensorgrams indicated slow association kinetics (Figure S4, Supporting Information), which likely accounts for the lack of observable binding in ITC. Overall, these results indicate that multiple active compounds recovered by iterative screening bind directly to RNA motifs, as evidenced by orthogonal biophysical assays, and can therefore be considered validated RNA binders.

To gain further insight into the binding mode of a representative validated binder, compound **8**, to RNA motif 2, nuclear magnetic resonance (NMR) experiments were performed. NMR spectra of RNA motif 2 were recorded in the absence and presence of compound **8** (Figure S5, Supporting Information). In the imino proton region, signals in the 13.0–13.5 ppm range exhibited line broadening and chemical shift perturbations upon compound addition, while the overall spectral changes remained relatively moderate. In the H5–H6 correlations, several clear chemical shift perturbations and signal disappearances were observed. The observed pattern of spectral changes suggests that, although the impact on base pairing is limited, compound **8** interacts with nucleobases within a specific region of the RNA. Compound **8** is therefore unlikely to bind to RNA motif 2 through nonspecific interactions with stem grooves or base‐pair planes. Instead, the perturbations are consistent with interactions involving loop residues. Taken together with the increase in melting temperature observed in the qFRET assay, these NMR results support binding of compound **8** to a localized region consistent with the internal loop of RNA motif 2.

### 2.5 Pharmacophore‐Guided Analog Design

**FIGURE 5:**
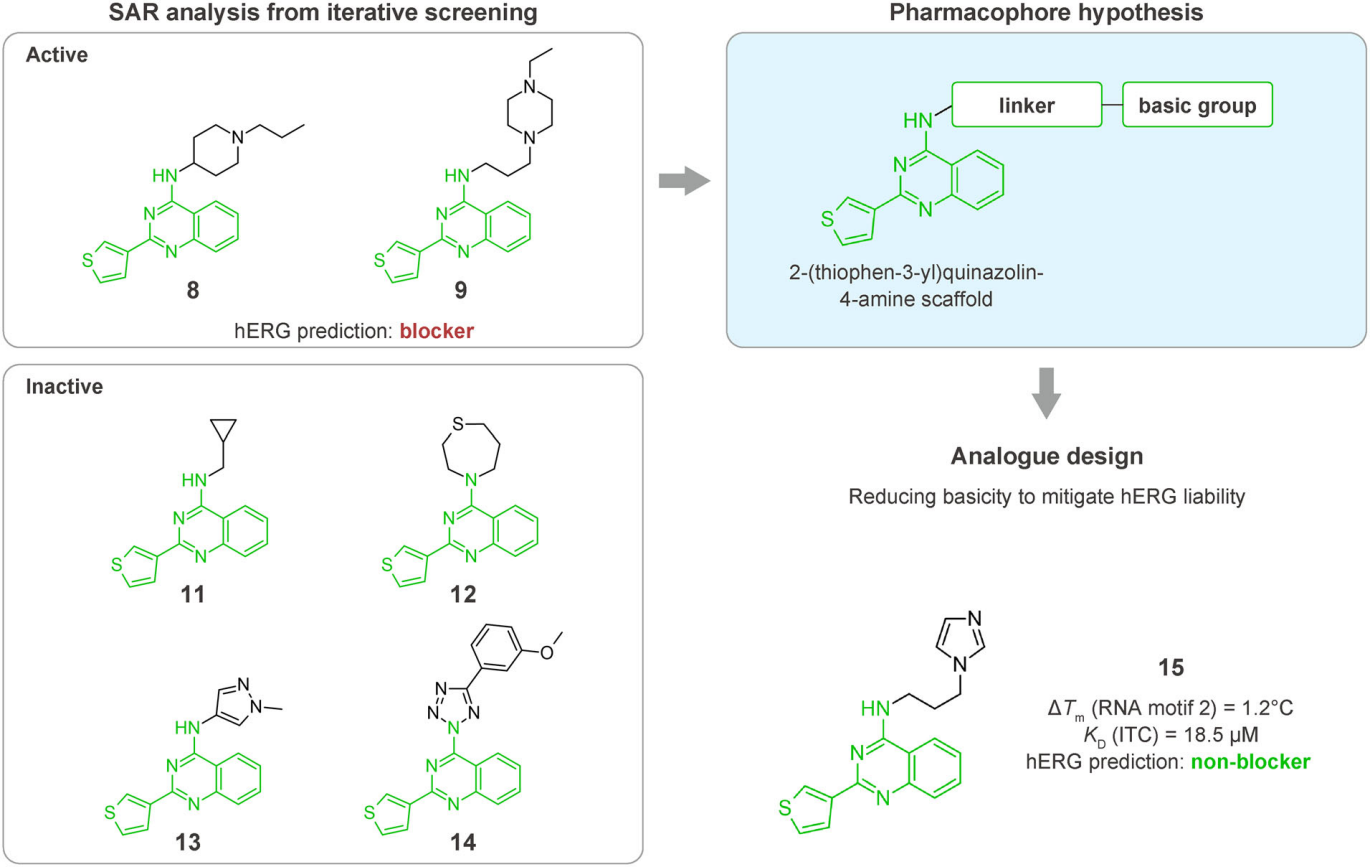
Structure–activity relationship (SAR) analysis from iterative screening that lead to the rational design of compound **15**. In silico prediction of human *Ether‐à‐go‐go‐*Related Gene (hERG) inhibition was performed using the Pred‐hERG webserver [48]. Simplified Molecular Input Line Entry System (SMILES) representations of the test compounds were submitted to the platform (http://labmol.com.br/predherg/), which provides a binary classification output (blocker or nonblocker) based on consensus machine learning models. These predictions were used to evaluate the potential cardiotoxicity of the compounds via hERG channel blockade.

We next examined the SAR tractability of compounds **8** and **9** to derive a pharmacophore hypothesis, a crucial step for rational analog design (Figure 5). Compounds sharing a common substructure (green) with compounds **8** and **9** were selected from those generated up to the sixth iteration. Through this process, compounds **11** to **14** were identified; however, these analogs were inactive. Comparison of the structures of the actives and inactives enabled the identification of structural features associated with activity retention, leading to the proposal of a pharmacophore hypothesis. Notably, an amino moiety conjugated via a linker to a 2‐(thiophen‐3‐yl)quinazolin‐4‐amine scaffold was identified as important for maintaining activity.

In parallel with activity‐driven SAR analysis, early safety‐related properties were also considered. Compounds **8** and **9** were predicted to be potential human *Ether*‐*à*‐*go*‐*go*‐Related Gene (hERG) channel blockers. Because hERG channel blockade is a known cause of cardiotoxicity [49, 50], evaluation of hERG liability during hit validation is recognized as an important step [48, 51, 52]. The presence of strongly basic nitrogen atoms is known to increase the risk of hERG inhibition [48]. Therefore, it has been suggested that lowering basicity can contribute to reduced hERG liability. Based on this consideration, compound **15** was designed by replacing the 4‐ethylpiperazine moiety of compound **9** with an imidazole moiety, with the goal of retaining RNA‐binding activity while reducing basicity.

Encouragingly, compound **15** exhibited qFRET activity and RNA motif 2 binding in ITC, with a *K*_D_ comparable to that of compound **9** (Figure S6, Supporting Information). Moreover, compound **15** was predicted to have lower hERG liability than compounds **8** and **9**, suggesting a potentially improved safety profile. Collectively, these findings suggest that SAR information derived from iterative screening can provide a foundation for pharmacophore hypothesis generation and support rational analog design.

### 2.6 Structural Insights into RNA Binding

To gain structural insight into the binding of small molecules to RNA motif 2, we first determined the X‐ray crystal structure of a pseudo‐self‐complementary (PSC) RNA construct designed to fold into a duplex containing two copies of the internal loop of RNA motif 2 (Figure S7, Supporting Information). RNA internal loops are generally characterized by high conformational flexibility, and multiple metastable conformations are known to coexist [53]. Consistent with this property, the crystal structure (PDB ID: 9VSN) revealed variability in base arrangements around the internal loop, with no single conformation clearly predominating.

**FIGURE 6:**
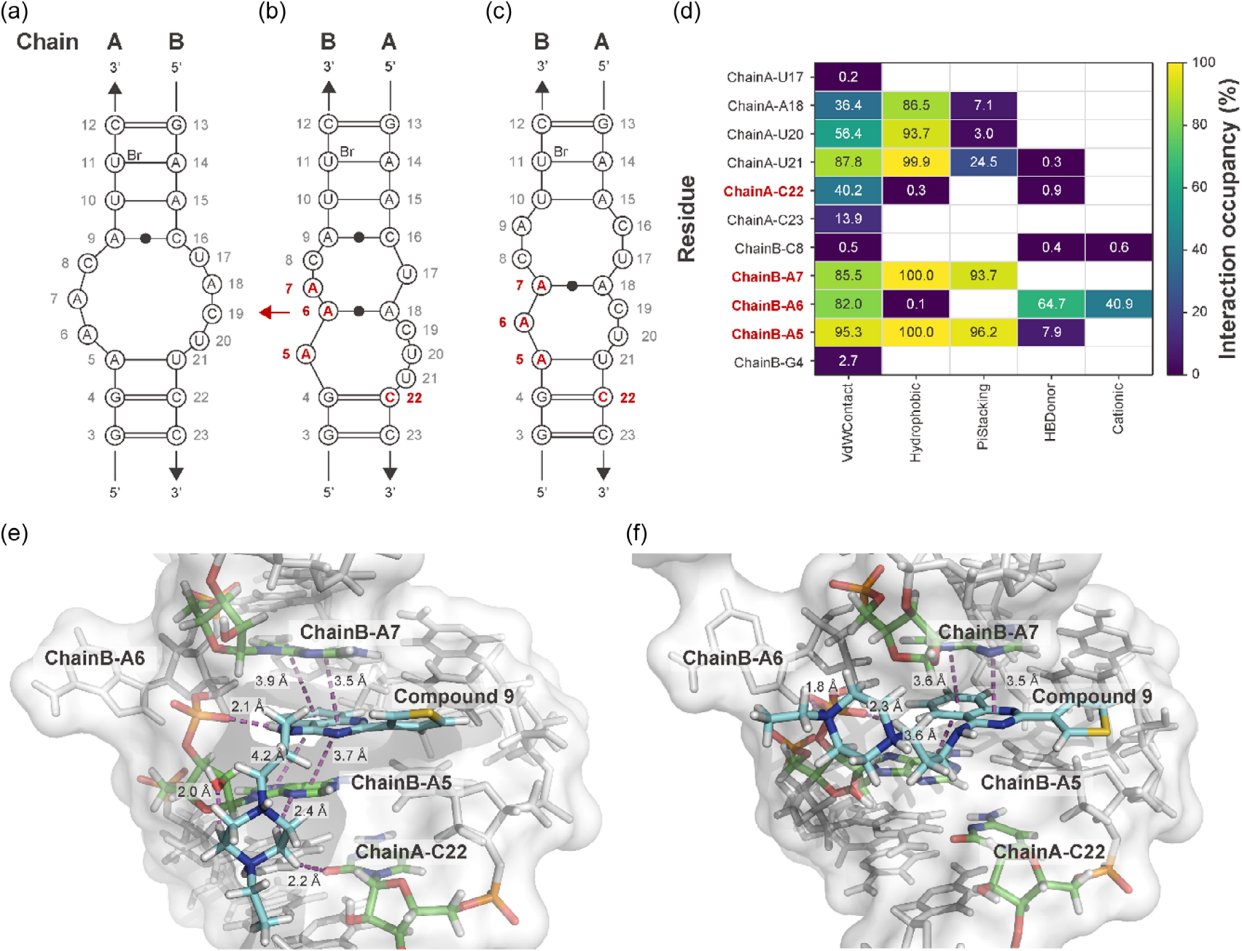
Predicted binding mode of compound **9** to RNA motif 2 based on docking and molecular dynamics (MD) simulations. (a–c) Secondary structure representations of the internal loop of RNA motif 2 within the pseudo‐self‐complementary (PSC) RNA duplex crystal structure (PDB ID: 9VSN). (a) First internal loop. (b) Second internal loop; the A6 residue of chain B, which forms a weak non‐Watson–Crick A:A base pair, was flipped out to generate the docking template. (c) Second internal loop after docking of compound **9** followed by MD simulations. (d) Interaction‐occupancy heatmap derived from ProLIF analysis of the selected MD trajectory (run03; Figures S7 and S8, Supporting Information). Rows correspond to RNA residues from chains A and B, and columns represent distinct interaction types (van der Waals contacts, hydrophobic interactions, π–π stacking, hydrogen‐bond donor interactions, and cationic interactions). Values indicate the percentage of simulation frames in which each interaction was detected; white cells denote 0% occupancy. Residues predicted to interact with compound **9** in the docking model are shown in red. Residue C19 from chain A is not included in the heatmap because it remains distal from the ligand throughout the MD trajectory and is not expected to form detectable interactions. (e, f) Representative MD snapshots showing the binding poses of compound **9** within the internal loop, highlighting hydrogen‐bond donor interactions, π–π stacking interactions, cationic interactions, and distances between interacting atoms from (e) the most prevalent cluster and (f) the second most prevalent cluster.

Crystallographic analyses of ribosomal RNA have shown that adenine bases in internal loop and bulge regions frequently adopt flipped‐out conformations [54, 55]. In light of this intrinsic structural plasticity, we employed a docking strategy in which an adenine residue within the internal loop was initialized in a flipped‐out orientation, enabling systematic exploration of binding conformations compatible with the observed flexibility of the RNA [56] (Figure 6a–c).

The resulting RNA–ligand complexes were subjected to all‐atom molecular dynamics (MD) simulations in explicit solvent. RNA was modeled using the YIL RNA force field [57], and ligand parameters were generated in a manner consistent with the same force‐field framework. Each complex was solvated in a cubic box of TIP3P water with a minimum solute–box distance of 12.0 Å, and Na^+^ counterions were added to neutralize the system charge without introducing excess salt. After equilibration, production MD simulations were carried out in the NPT ensemble at 310.15 K and 1 bar for 100 ns using a 0.5 fs time step. To account for the intrinsic conformational heterogeneity of RNA internal loops, five independent replicas were performed for each docked complex, differing only in their initial velocity assignments (Figures S8 and S9, Supporting Information).

To identify a representative ligand‐bound conformation, trajectories from the independent MD simulations were first compared with respect to overall structural stability of the RNA–ligand complex. One trajectory exhibiting consistently reduced global deviations and suppressed local fluctuations of the RNA internal loop was selected for detailed analysis. RNA–ligand interactions were analyzed using interaction fingerprint analysis implemented in ProLIF [58], focusing on noncovalent interactions between the ligand and nucleotides in the internal loop region. This trajectory displayed persistent aromatic stacking interactions between the ligand and the RNA, with stable π–π stacking against two adenine residues (ChainB‐A5 and ChainB‐A7) in the internal loop. These interactions were accompanied by moderately persistent hydrogen bonding and cationic interactions with a nearby phosphate group (ChainB‐A6), together defining a reproducible and spatially organized binding geometry.

The observed cationic interaction between compound **9** and ChainB‐A6 is consistent with our pharmacophore hypothesis, which suggests a role for the presence of a basic group (Figure 6d). The representative binding pose of the most prevalent cluster is shown in Figure 6e, while that of the second most prevalent cluster is shown in Figure 6f. In both poses, the 2‐(thiophen‐3‐yl)quinazoline scaffold engages in π–π stacking interactions. In the binding pose shown as Figure 6e, the adjacent NH group forms a hydrogen bond with the phosphate backbone of ChainB‐A6. In contrast, the linker and terminal amino group extend outward from the binding pocket, suggesting potential involvement in additional interactions with residues such as ChainB‐A5 (Figure 6e) or ChainB‐A6 (Figure 6f).

To further assess the stability of the proposed binding pose, five additional independent short MD simulations (10 ns duration each) were performed from the binding pose shown in Figure 6e (Figures S10–S12, Supporting Information). Across all replicas, the RNA–ligand complex remained structurally stable as assessed by root‐mean‐square deviation (RMSD), and the same key stacking and hydrogen‐bonding interactions were consistently preserved. The observed structural stability of the selected ligand‐bound conformation suggests that this binding mode is both stable and reproducible under the simulation conditions. On this basis, the identified interaction pattern represents a plausible model for ligand–RNA recognition. These observations are consistent with the SAR trends derived from iterative screening and support the proposed pharmacophore hypothesis.

We note that the RNA crystal structure used for docking was determined at 3.1 Å resolution. At this resolution, precise orientations of nucleobases, phosphate groups, and hydrogen‐bonding geometries cannot be assigned with atomic accuracy, and water‐ or ion‐mediated interactions cannot be reliably resolved. Accordingly, the docking and MD analyses are interpreted in terms of recurrent interaction patterns and spatial proximity rather than specific hydrogen‐bond geometries or energetic contributions.

## 3 Conclusion

In summary, we developed AISLAR as a workflow for discovering RNA‐targeted small molecules. Using chemically diverse libraries and the open‐source KNIME platform, AISLAR enabled efficient recovery of chemotypes amenable to SAR analysis. Early SAR patterns informed the generation of a pharmacophore hypothesis, which in turn supported rational analog design. The X‐ray crystal structure of a construct containing the internal loop of RNA motif 2 provided a structural framework for docking studies, offering mechanistic insight into RNA–ligand interactions. Notably, the overall effectiveness of AISLAR was contingent on reliable screening data; in this case, the accuracy of the qFRET assay was critical for hit triage and SAR analysis.

This work demonstrates that screening data alone can guide assessments of SAR tractability, allowing chemotypes with higher optimization potential to be prioritized early. AISLAR improved the efficiency of screening for RNA‐targeted small molecules by recovering high‐quality hits after sampling only a fraction of the full library. Furthermore, identifying positions tolerant to chemical modification creates opportunities for functionalizing RNA ligands into degraders or covalent binders, which have attracted increasing interest in recent years [59, 60, 61]. With its flexible workflow architecture and reliance on open‐source tools, AISLAR can, in principle, be adapted to different structured RNA motifs and implemented with alternative assay formats. While further validation across diverse RNA targets and compound libraries will be required, this flexibility enables adaptation to RNA targets with differing structural features and dynamics, thereby providing a general framework for RNA‐targeted small molecule discovery.

## 4 Experimental Section/Methods

### 4.1 Chemicals, Compound Libraries, and RNA

High‐performance liquid chromatography‐purified RNA oligonucleotides representing RNA motifs 1 and 2, as well as the crystallization construct were purchased from Hokkaido System Science Co., Ltd. (Sapporo, Japan). These included unlabeled, 5′‐biotinylated, and dual‐labeled (5′‐Cy5 and 3′‐BHQ2) RNAs, as well as the brominated RNA crystallization construct. Screening was performed using the FDA‐approved drug library from MedChemExpress (Monmouth Junction, NJ, USA), and the DDS and RDS from Enamine (Kyiv, Ukraine). Compound **15** was purchased from Enamine (Kyiv, Ukraine).

### 4.2 RNA Secondary Structure Prediction and Motif Selection

The secondary structures of human p53 mRNA (RefSeq: NM_000546.6) were predicted using the RNAfold Web Server from the ViennaRNA Package with default parameters [62]. The minimum free energy structure revealed multiple stem‐loops.

A stem‐loop spanning nucleotides U187 to A225 was selected as RNA motif 2 (Figures 1 and S1, Supporting Information). A second stem‐loop derived from a different region of the p53 mRNA sequence was selected as RNA motif 1 (Figures 1 and S1, Supporting Information). RNA motif 1 features a distinct internal‐loop geometry relative to RNA motif 2 and was used as an additional RNA probe for selectivity assessment.

### 4.3 Screening Using qFRET

Thermal shift assays based on qFRET were employed to assess ligand‐induced stabilization of fluorescently labeled RNA probes corresponding to RNA motifs 1 and 2 [5]. Reaction mixtures (10 μL), containing 50 nM dual‐labeled RNA, 0.1 mg mL^−1^ bovine serum albumin (Takara Bio, Kusatsu, Japan), 20 mM potassium phosphate buffer, pH 6.5, 50 mM potassium chloride, 1% dimethyl sulfoxide (DMSO), and 100 μM compound, were prepared in 384‐well polymerase chain reaction (PCR) plates. Cy5 fluorescence (λ_ex = 648 nm, λ_em = 668 nm) was monitored using a CFX384 Touch Real‐Time PCR Detection System (Bio‐Rad, Hercules, CA).

The thermal protocol comprised an initial denaturation step at 95°C for 15 s and a cooling step at 4°C for 2 min, followed by temperature increments of 2°C up to 95°C from 25°C, with fluorescence readings taken after 10 s at each temperature. Melting temperatures (*T*_m_) were derived from the second derivative of the fluorescence curves, and the Δ*T*_m_ values were calculated as the difference between *T*_m_ in the presence and absence of compound.

### 4.4 Machine Learning Workflow and Performance Evaluation

#### 4.4.1 Data Preparation

Compounds lacking reliable Δ*T*_m_ values for either RNA motif 1 or 2 were excluded from the dataset, including cases where the melting curves were irregular, nonsigmoidal, or lacked a well‐defined inflection point required for robust Δ*T*_m_ determination.

#### 4.4.2 Hyperparameter Optimization and Model Training

Hyperparameter optimization was performed using the H2O.ai platform (H2O‐3, version 3.46.0.1) via the AutoML module of the KNIME Analytics Platform (version 5.4.0). A total of 7,165 data points from the full qFRET HTS dataset for RNA motifs 1 and 2 were independently used for hyperparameter optimization (Table S1, Supporting Information). The algorithms evaluated included Random Forest, with the maximum relative number of rows after balancing defined as 1, and all other parameters left at default values. The best performing models for both datasets were Random Forest classifiers with 50 trees and a maximum depth of 20, selected based on the cross‐validated Area Under the Precision–Recall Curve.

The optimized hyperparameters were subsequently used to train the initial model based on the results of the first iteration. This model was then applied to predict the activity of the remaining untested compounds. EFs were calculated and plotted as a function of the number of active compounds in the top x% of predicted scores (Figure S2, Supporting Information). For EF calculation, the total number of compounds and the total number of actives were defined as those in the full library, including compounds previously tested. Selecting the top 5% of predicted compounds resulted in EFs of 5.5 for RNA motif 1 and 6.5 for RNA motif 2, indicating substantially higher active recovery than random selection. Because the EFs were considered sufficient for demonstrating usefulness of iterative screening, the iteration size was set to 5%.

EF was calculated as

(1) EF=(Actives_sample/N_sample)/(Actives_total/N_total)

where Actives_sample is the number of compounds classified as actives based on the qFRET screening criterion within the sample, N_sample is the number of compounds in the sample, Actives_total is the number of compounds classified as actives based on the qFRET screening criterion within the full dataset, and N_total is total number of compounds.

To ensure reproducibility, the KNIME workflow used in iterative screening simulations is included in the Supporting Information (KNIME_workflow.knwf). An example input file (WorkflowInput_ExampleSubset.csv), containing the 14 representative compounds, is also provided to illustrate the required file format.

#### 4.4.3 Classification Metrics and Model Performance

To characterize Random Forest classification performance, we computed the precision, recall, and F1‐score to characterize using experimentally determined qFRET activity as ground truth (Table S2, Supporting Information). Compounds were labeled as active or inactive based on the experimental criterion described above (Δ*T*_m_ > 1°C). Precision was defined as the fraction of Random Forest‐predicted active compounds that were experimentally active, recall as the fraction of experimentally actives recovered by the model, and F1‐score as the harmonic mean of precision and recall. These metrics provide a complementary assessment of model performance under the strongly imbalanced distribution observed in the primary screen.

#### 4.4.4 Selectivity Analysis and Chemical Space Visualization

For each RNA motif, the number of active compounds recovered by iterative screening that also showed activity against the other probe was quantified. The overlap between motif‐preferential and shared actives was visualized to illustrate selectivity patterns and inclusion relationships for models optimized on RNA motif 1 or RNA motif 2 (Figure S3, Supporting Information).

Chemical space visualization was performed using UMAP generated in Datawarrior (version 06.04.02). Embeddings were computed from ECFP4 fingerprints generated using the RDKit package.

### 4.5 BLI Measurements

BLI experiments were performed using the Octet RH16 System (Sartorius AG, Göttingen, Germany) in black, 384‐well microplates (Octet 384 Well Tilted Bottom Plate, Sartorius AG) at 30°C. All measurements were carried out in BLI buffer consisting of 20 mM potassium phosphate, pH 6.5, 50 mM potassium chloride, 50 mM sodium chloride, and 0.05% Tween 20 containing 5.0% DMSO.

To immobilize RNA, super streptavidin biosensors (Sartorius AG) were first equilibrated in immobilization buffer consisting of 20 mM potassium phosphate, pH 6.5, 50 mM potassium chloride, 500 mM sodium chloride, 0.05% Tween 20, and 10% DMSO for 60 s, then loaded with 5′‐biotinylated RNA motif 1 or 2 (500 nM) in immobilization buffer for 600 s. Compounds stocked as 10 mM DMSO solutions were diluted to 2.5 mM with DMSO, followed by 1:2 serial dilutions and the addition of BLI buffer containing 1% DMSO to each resultant solution, yielding test compound solutions at eight concentrations (0.8–100 μM).

Binding measurements were performed in ascending order of concentration using the following sequence: 600 s delay, 60 s baseline, 60 s association, and 60 s dissociation. Reference controls were used for subtraction: RNA unloaded reference biosensors exposed to the same compound series. Raw data were analyzed using Octet Analysis Studio 12.2 (Sartorius AG). Sensorgrams were processed by reference subtraction, and kinetic parameters (*k*_a_, *k*_d_, and *K*_D_) were obtained by fitting into a 1:1 binding model.

### 4.6 ITC Measurements

ITC experiments were conducted at 25°C using the MicroCal PEAQ‐ITC instrument (Malvern Panalytical Ltd., Worcestershire, UK). RNA motif 2 and compounds were dissolved in ITC buffer (20 mM potassium phosphate, pH 6.5, 50 mM potassium chloride, 2% DMSO). The sample cell was loaded with 10 μM RNA, and titrations were performed with 200 μM compound.

Each titration began with a 0.4 μL injection followed by 15 or 16 injections of 2.5 μL, spaced 100 s apart. The first injection was excluded from analysis. Control titrations of ligand into buffer were performed to account for dilution heat, which was subtracted from the experimental data. Thermograms were analyzed using the MicroCal PEAQ‐ITC Analysis software and fitted into a one‐site binding model to extract Δ*H*, N, and *K*_D_.

### 4.7 NMR Spectrometry

RNA motif 2 and compound **8** were prepared in NMR buffer (20 mM potassium phosphate buffer, pH 6.5, 50 mM potassium chloride, 5% D_2_O) at a final concentration of 40 μM containing 0.4% DMSO. NMR measurements were performed on a Bruker AVANCE III HD 600 spectrometer (Bruker BioSpin GmbH, Rheinstetten, Germany) equipped with a cryo‐TXI probe at the RIKEN Yokohama NMR Facility at 288K. One‐dimensional ^1^H spectra were collected for using the p11 jump‐and‐return water suppression pulse sequence. Two‐dimensional ^1^H–^1^H total correlation spectroscopy spectra were collected using the mlevgpph19 pulse sequence. NMR data were processed using the TopSpin 3.6 software (Bruker BioSpin).

### 4.8 Crystallization, X‐Ray Data Collection, and Structure Determination

For crystallographic analysis, we used a PSC RNA construct that was designed to fold into a duplex containing two copies of the internal loop within RNA motif 2 (Figure S6) [63, 64]. Two 5′‐terminal uridine residues were added to facilitate crystal packing.

Crystallization was performed at 293 K by the hanging‐drop vapor diffusion method. Each drop consisted of 1 μL of 1 mM RNA and 1 μL of crystallization solution from an in‐house screening kit, equilibrated against 250 μL of reservoir solution. Crystals of PSC were obtained in a condition containing 50 mM sodium cacodylate (pH 7.0), 10 mM spermine tetrahydrochloride, 400 mM potassium chloride, and 10% (v/v) 2‐methyl‐2,4‐pentanediol. Crystals were briefly soaked in cryoprotectant solution (40% v/v 2‐methyl‐2,4‐pentanediol) and flash‐cooled in liquid nitrogen.

X‐ray diffraction data for one of the PSC crystals were collected at 100 K using synchrotron radiation on beamline BL‐17A at the Photon Factory (Tsukuba, Japan). The data were processed using *XDS* [65], and the structure was solved by molecular replacement with *Phaser* (*Phenix* suite) [66, 67], using published ribosomal A‐site structures as search models. Model building was carried out using *Coot* [68, 69], and refinement was carried out using *phenix.refine*, employing simulated annealing, gradient minimization, and B‐factor refinement [66, 70]. Data collection and refinement statistics are provided in Table S3 (Supporting Information). Molecular graphics were prepared using *PyMOL* [71], and the coordinates and structure factors have been deposited in the Protein Data Bank under accession code 9VSN.

### 4.9 Docking and MD Simulations

#### 4.9.1 Software and Computational Environment

Docking calculations were performed using RxDock [72] (rbcavity, rbdock; version 0.1.0) in a Linux‐based environment. Docking parameters and execution commands are described below.

MD simulations were performed using GROMACS [73] version 2024.5. RNA–ligand interaction analyses were carried out using ProLIF (version 2.0.3) implemented in Python, in combination with RDKit (version 2025.09.3) for ligand handling and MDAnalysis (version 2.10.0) for trajectory processing. All analyses were executed in a conda‐managed environment to ensure reproducibility.

#### 4.9.2 Generation of Flipped‐Out Adenine Starting Conformations

To generate initial models in which an adenine base within the internal loop is oriented in a flipped‐out conformation, a rigid‐body rotation was applied to a local nucleotide segment using a custom Python script. Briefly, a rotation axis was defined by the line connecting the O3′ atom of residue i − 1 and the O5′ atom of residue i + 1 (PDB residue numbering was used; insertion codes, when present, were handled explicitly). All atoms of residue i were rotated as a rigid body about this axis by an incremental angle Δ (degrees), while the phosphate group of residue i + 1 (P, OP1, and OP2) was rotated concomitantly to preserve local backbone continuity. The rotation was implemented using a standard axis–angle rotation matrix and applied to Cartesian coordinates of the selected atoms. Rotations were performed on the apo RNA crystal structure to generate a series of flipped‐out starting conformations spanning the desired angular range, and the resulting coordinates were written as PDB files for subsequent docking.

#### 4.9.3 Molecular Docking Using RxDock

Docking was performed using RxDock to explore small‐molecule binding poses to the flipped‐out internal loop conformation of the PSC RNA construct. The receptor structure was provided in MOL2 format, and docking parameters were specified using an RxDock parameter file (RBT_PARAMETER_FILE_V1.00). The binding site was defined using the RbtSphereSiteMapper, with the site center set to (16.27, −12.81, −15.92) and a radius of 10.0 Å, allowing identification of a single cavity. Receptor flexibility was enabled with a tolerance of 3.0 Å.

Cavity evaluation was performed using the RbtCavityGridSF scoring function. In addition, pharmacophore‐based restraints were incorporated via RbtPharmaSF, with constraints defined in an external file. The pharmacophore model comprised hydrophobic/aromatic (Har), hydrogen‐bond acceptor (Acc), and cationic (Cat) features positioned at predefined coordinates, each assigned a unit weight. The cavity and pharmacophore scoring terms were applied with equal weights during docking.

Docking was carried out using the SF5 scoring function, which explicitly includes a desolvation potential. Docking grids were generated using the rbcavity module, and ligand docking was performed with rbdock using 50 independent runs per ligand to ensure sufficient conformational sampling.

#### 4.9.4 Force Fields and System Preparation

RNA was modeled using the YIL RNA force field [57], while ligand parameters were generated consistently with the same force‐field framework. The RNA–ligand complex was solvated in a TIP3P water model using a cubic simulation box with a minimum solute–box distance of 12.0 Å (TIP3PBOX, isotropic). Counterions (Na^+^) were added at random positions to neutralize the total system charge (addIonsRand, Na^+^ only), resulting in a neutral system without additional excess salt.

#### 4.9.5 Energy Minimization and Equilibration

Energy minimization was performed using the steepest descent algorithm to remove steric clashes and unfavorable contacts. The minimization was terminated when the maximum force fell below 0.0418 kJ mol^−1^ nm^−1^, or after a maximum of 50,000 steps. Long‐range electrostatic interactions were treated using the Particle Mesh Ewald method, with short‐range electrostatic and van der Waals cutoffs set to 1.2 nm.

During the early stages of equilibration, positional restraints were applied to stabilize the RNA–ligand complex. Restraints were applied to heavy atoms of both the RNA and ligand, with additional restraints on the RNA backbone. These restraints were gradually released during subsequent equilibration steps.

Equilibration was carried out in multiple stages, consisting of NVT and NPT simulations with progressively relaxed restraints. All MD simulations used a leap‐frog integrator with a time step of 0.5 fs. Temperature was maintained at 310.15 K using the velocity‐rescaling (V‐rescale) thermostat, and pressure was maintained at 1.0 bar using the C‐rescale barostat with isotropic coupling.

Electrostatic interactions were treated using the Particle Mesh Ewald method. The final equilibration stage consisted of a 1 ns unrestrained NPT simulation to ensure stable temperature, pressure, and density prior to production runs.

#### 4.9.6 Production MD Simulations

Production MD simulations were performed in the NPT ensemble at 310.15 K and 1 bar, using the same thermostat and barostat settings as in equilibration. Each production run was conducted for 100 ns with a 0.5 fs time step. Coordinates were saved every 10 ps in compressed trajectory format, and energies and log files were written every 1 ps.

To assess the robustness of sampling and account for the intrinsic conformational heterogeneity of RNA internal loops, five independent MD replicas were performed for each docked complex. Each replica was initiated with different randomized initial velocities, while all other simulation parameters were kept identical.

#### 4.9.7 Interaction Analysis and Selection of a Representative Ligand‐Bound Conformation

MD trajectories were analyzed to evaluate ligand stability and RNA–ligand interactions. RNA–ligand interaction fingerprints were generated using ProLIF, focusing on noncovalent contacts involving nucleotides in the internal loop region.

Across the independent replicas, several trajectories exhibited stable ligand positioning within the internal loop. One representative trajectory displaying persistent RNA–ligand interactions and limited ligand positional drift was selected for detailed analysis. In this trajectory, π–π stacking interactions with adenine residues in the internal loop and hydrogen‐bonding interactions with the RNA backbone were observed, along with additional transient contacts involving neighboring nucleotides.

Snapshots extracted from this trajectory were subjected to clustering analysis based on the RMSD of the ligand and surrounding RNA residues. The most populated cluster was identified as representing the dominant binding pose, and its centroid structure was selected as the representative ligand‐bound conformation for visualization and interpretation.

## Supporting Information

Additional supporting information can be found online in the Supporting Information section.

## Author Contributions

**Haruhiko Hattori**: conceptualization (equal), data curation (lead); formal analysis (lead); methodology (lead); validation (lead); visualization (equal); writing – original draft (lead); writing – review & editing (supporting). **Maina Otsu**: investigation (lead); writing – review & editing (supporting). **Koji Imai**: investigation (supporting); writing – review & editing (supporting). **Mebuki Narahara**: investigation (supporting); writing – review & editing (supporting). **Jiro Kondo**: investigation (supporting); writing – review & editing (supporting). **Amiu Shino**: conceptualization (equal); project administration (lead); supervision (equal); writing – review & editing (supporting). **Ella Czarina Morishita**: conceptualization (supporting); data curation (supporting); investigation (supporting); project administration (supporting); supervision (equal); validation (supporting); visualization (equal); writing – review & editing (lead).

## Conflicts of Interest

The authors declare no conflicts of interest.

## Supporting information

Supplementary Material

## Data Availability

The raw data supporting the findings of this study are not publicly available due to commercial restrictions. Detailed descriptions of the experimental procedures, data acquisition, and data processing workflows are provided in the Supporting Information to enable reproducibility. Researchers interested in accessing the raw data may contact the corresponding author, subject to appropriate confidentiality or data‐sharing agreements.
