# Supplementary material for: AI‐Augmented Iterative Screening of Libraries Against RNA Targets (AISLAR) Boosts Discovery of SAR‐Tractable RNA Binders and Rational Analog Design
Source: Small Sci. 2026 Apr 17;6(4):e202600007. doi: 10.1002/smsc.202600007 (PMC13088978; doi:10.1002/smsc.202600007)
Supplement: Supplementary file 1 — Supplementary Material [file SMSC-6-e202600007-s001.pdf]

Supporting Information

AI-Augmented Iterative Screening of Libraries against RNA Targets (AISLAR) Enables Discovery of SAR-Tractable Small-Molecule Binders

*Haruhiko Hattori, Maina Otsu, Koji Imai, Mebuki Narahara, Jiro Kondo, Amiu Shino, and Ella Czarina Morishita**

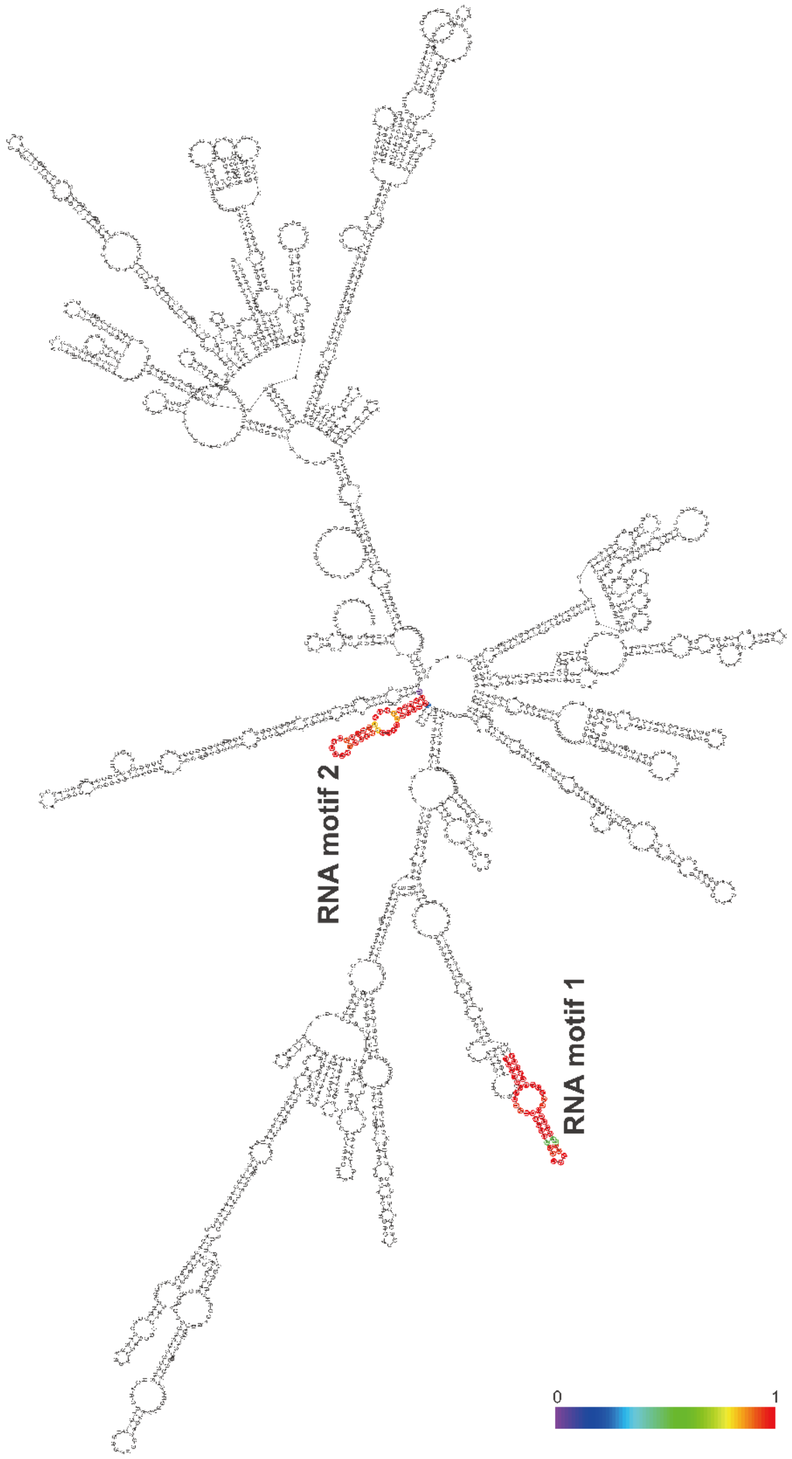

Figure S1. RNA secondary structure prediction of human p53 mRNA. The complete mRNA structure is shown, with RNA motifs 1 and 2 highlighted. For these motifs, base-pairing probabilities are overlaid to illustrate the stability and variability of predicted base pairs. Each predicted base pair is colored using a heatmap gradient from blue to red, representing base-pairing probabilities ranging from 0 to 1. Only residues within RNA motifs 1 and 2 are displayed with base-pairing probability information, while the remainder of the structure is shown schematically for context.

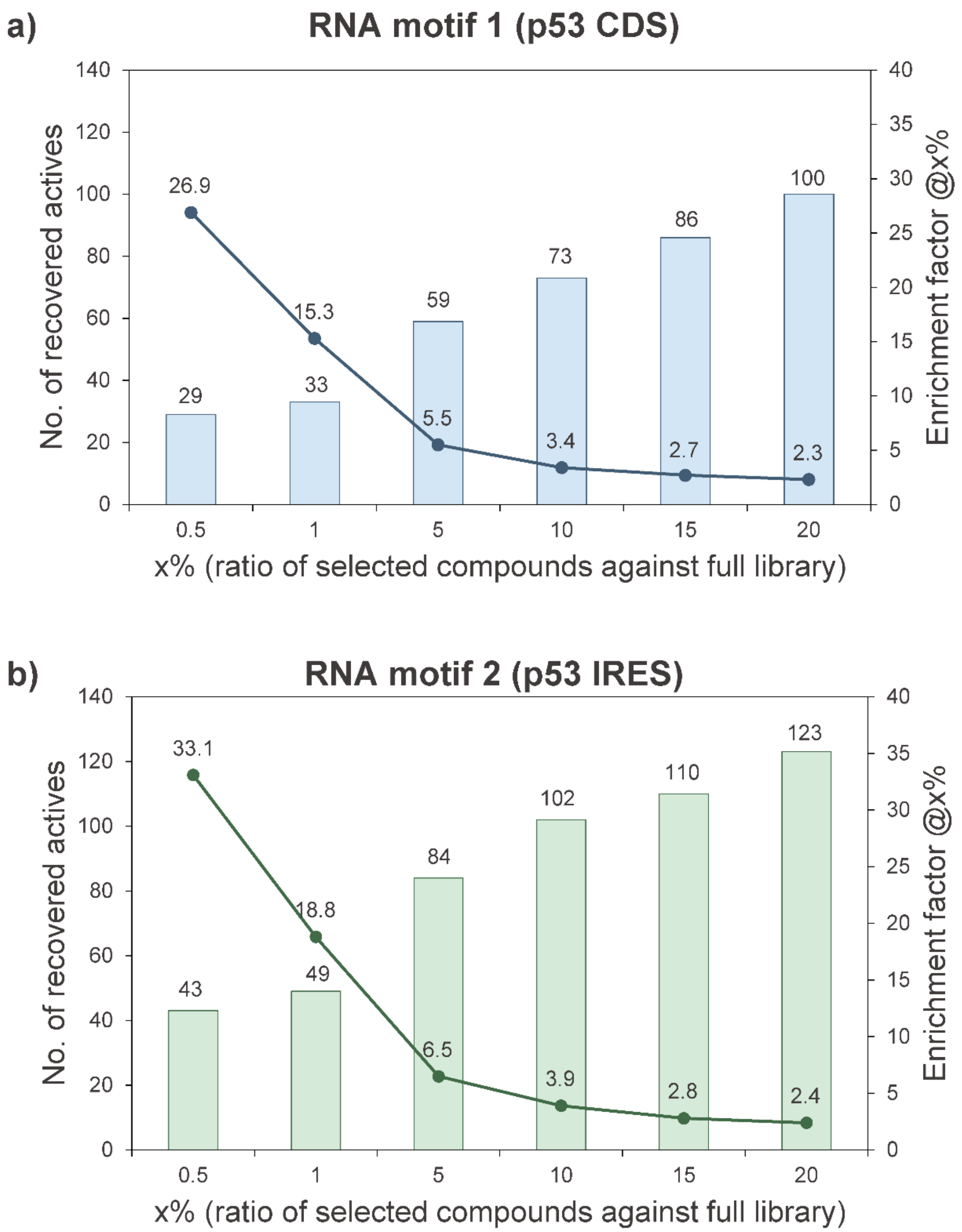

Figure S2. Evaluation of early recognition performance of the initial prediction models trained on first-iteration screening data for RNA motif 1 (a) and RNA motif 2 (b). The number of recovered active compounds within the top *x*% of ranked predictions (bars) and the corresponding enrichment factors (lines) are shown, illustrating early hit enrichment before iterative screening cycles.

Table S1. Ranking-based performance metrics of Random Forest models optimized using the first iteration subset for RNA motifs 1 and 2. Models were trained on 10% of the full compound library selected by RDkit diversity picker, with the Area Under the Precision-Recall Curve (AUPRC) used as the optimization objective. The Receiver Operating Characteristic Area Under the Curve (ROC-AUC) and the Enrichment Factor (EF) @5% are reported to evaluate overall ranking performance and early recognition performance, respectively. For reference, random ranking resulted in mean AUPRC values of 0.0051 ± 0.0034 and 0.0083 ± 0.0021 for RNA motifs 1 and 2, respectively, based on 100 random repeats.

Target	RNA motif 1	RNA motif 2
AUPRC	0.123	0.074
ROC-AUC	0.741	0.807
EF@5%	5.5	6.5

Table S2. Classification performance metrics for experimentally active compounds. Precision, recall, and F1-score were computed using experimentally determined qFRET activity as ground truth (see **Table 1** for the number and percentage of experimental actives). Values for random selection are shown as mean ± standard deviation over six independent results from random seeds. Low absolute precision values are expected due to the extreme class imbalance of the primary screen (≤1% actives). Accordingly, AISLAR is designed to prioritize early recovery of active compounds (recall and enrichment) rather than to function as a high-precision classifier.

Target	RNA motif 1 (AISLAR)	RNA motif1 (random)	RNA motif 2 (AISLAR)	RNA motif 2 (random)
Precision	0.0061	0.0029 ± 0.00031	0.0081	0.0036 ± 0.00024
Recall	0.71	0.33 ± 0.04	0.78	0.35 ± 0.02
F1-score	0.012	0.0057 ± 0.00062	0.016	0.0071 ± 0.00048

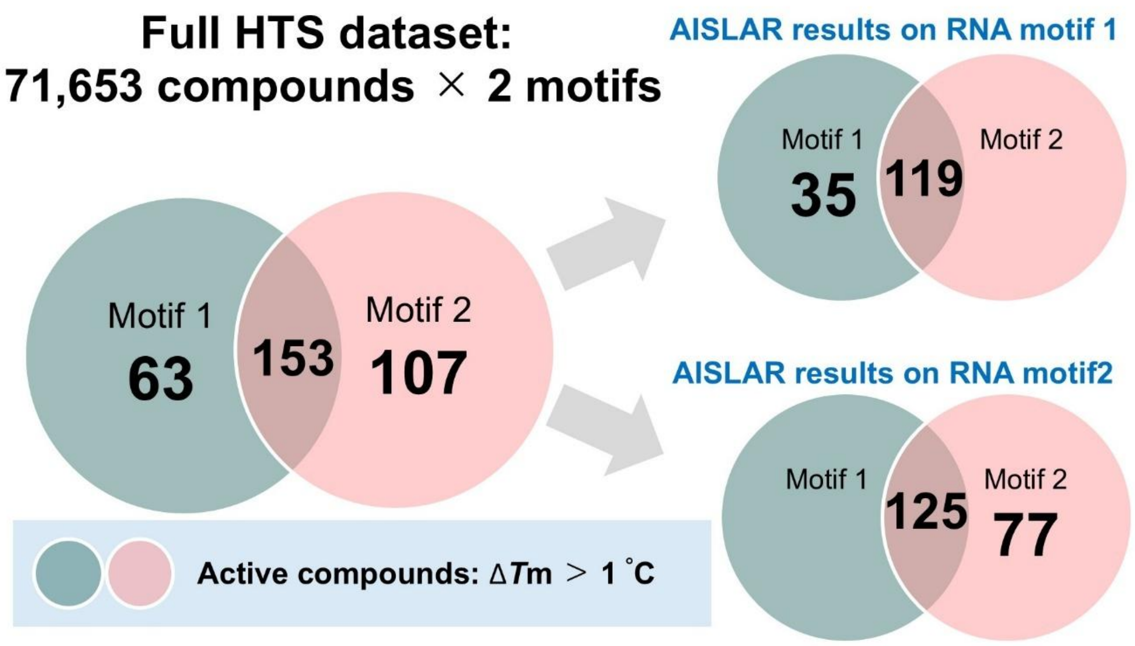

Figure S3. Selectivity patterns of active compounds in the full HTS dataset and among compounds recovered by AISLAR. Venn diagrams summarize the overlap of active compounds (ΔT_m > 1 °C) identified for RNA motif 1 and RNA motif 2. The left panel shows the distribution of actives in the full HTS dataset comprising 71,653 compounds screened against both RNA motifs. The right panels show subsets of active compounds recovered by AISLAR using machine learning models optimized independently on RNA motif 1 (top right) or RNA motif 2 (bottom right). Overlapping regions indicate compounds active against both RNA motifs, whereas non-overlapping regions represent motif-preferential actives. The comparable degree of overlap observed between the full HTS dataset and the AISLAR-recovered subsets indicates that iterative screening does not introduce additional bias toward broadly active or non-specific compounds.

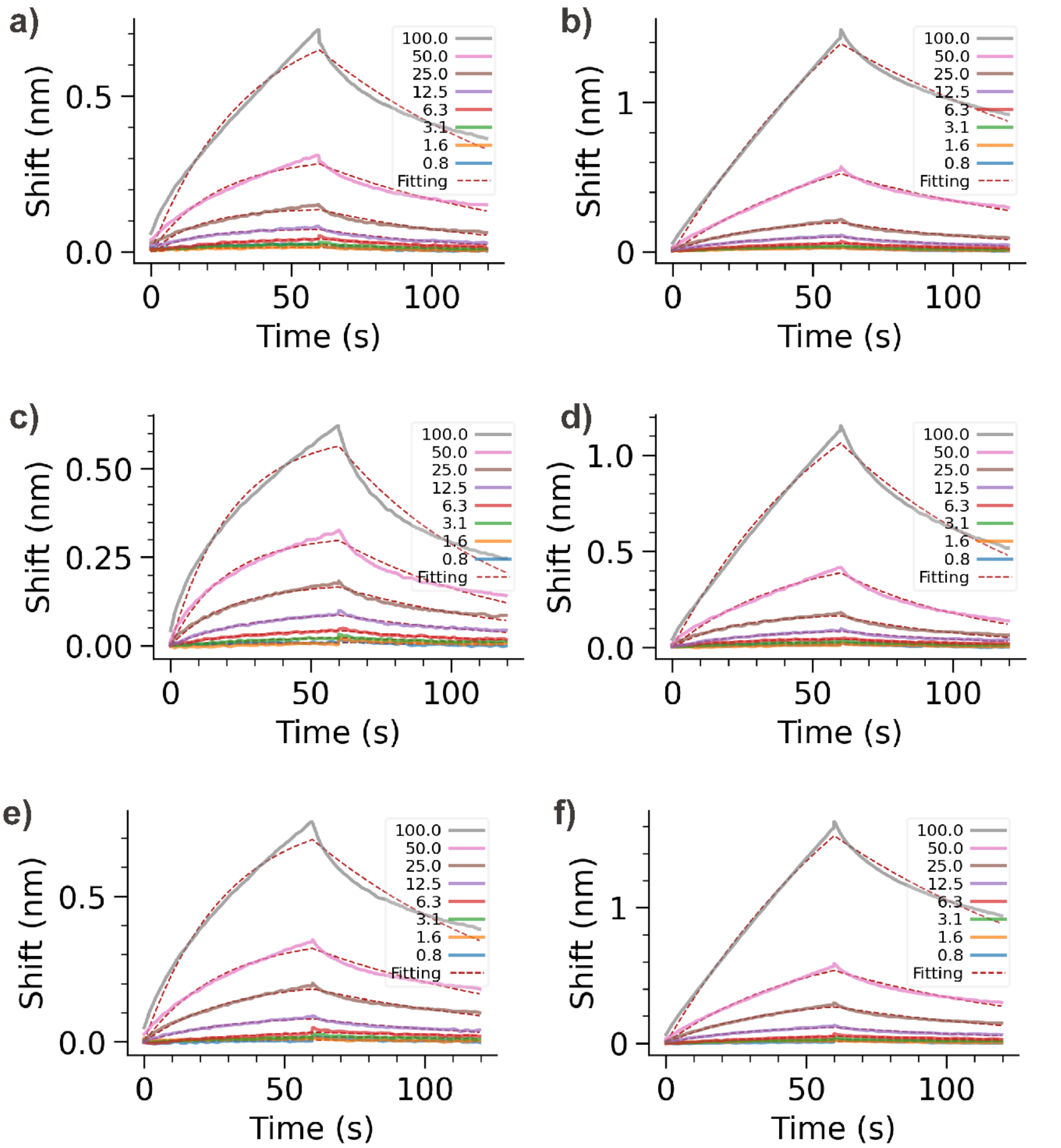

Figure S4. BLI sensorgrams showing RNA motif 1 binding with compound **1** (**a**), **2** (**c**), and **3** (**e**), and RNA motif 2 binding with compound **1** (**b**), **2** (**d**), and **3** (**f**).

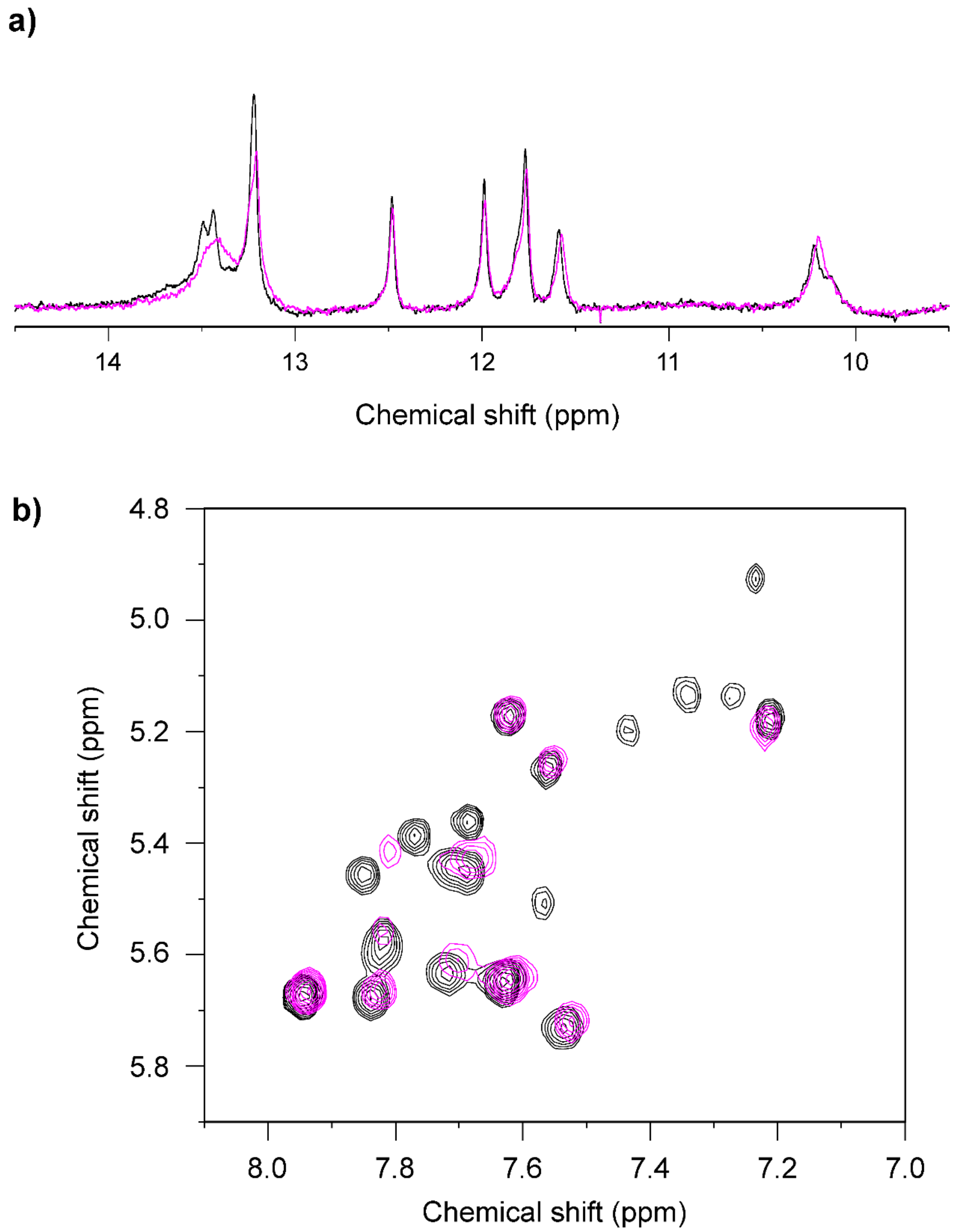

Figure S5. NMR spectra of RNA motif 2 alone (black) and RNA motif 2 in the presence of compound **8** (magenta), a close structural analogue of compound **9**. (**a**) One-dimensional ^{1}H NMR spectra for the exchangeable imino protons and (**b**) two-dimensional ^{1}H–^{1}H TOCSY spectra for H5–H6 correlation signals of pyrimidines.

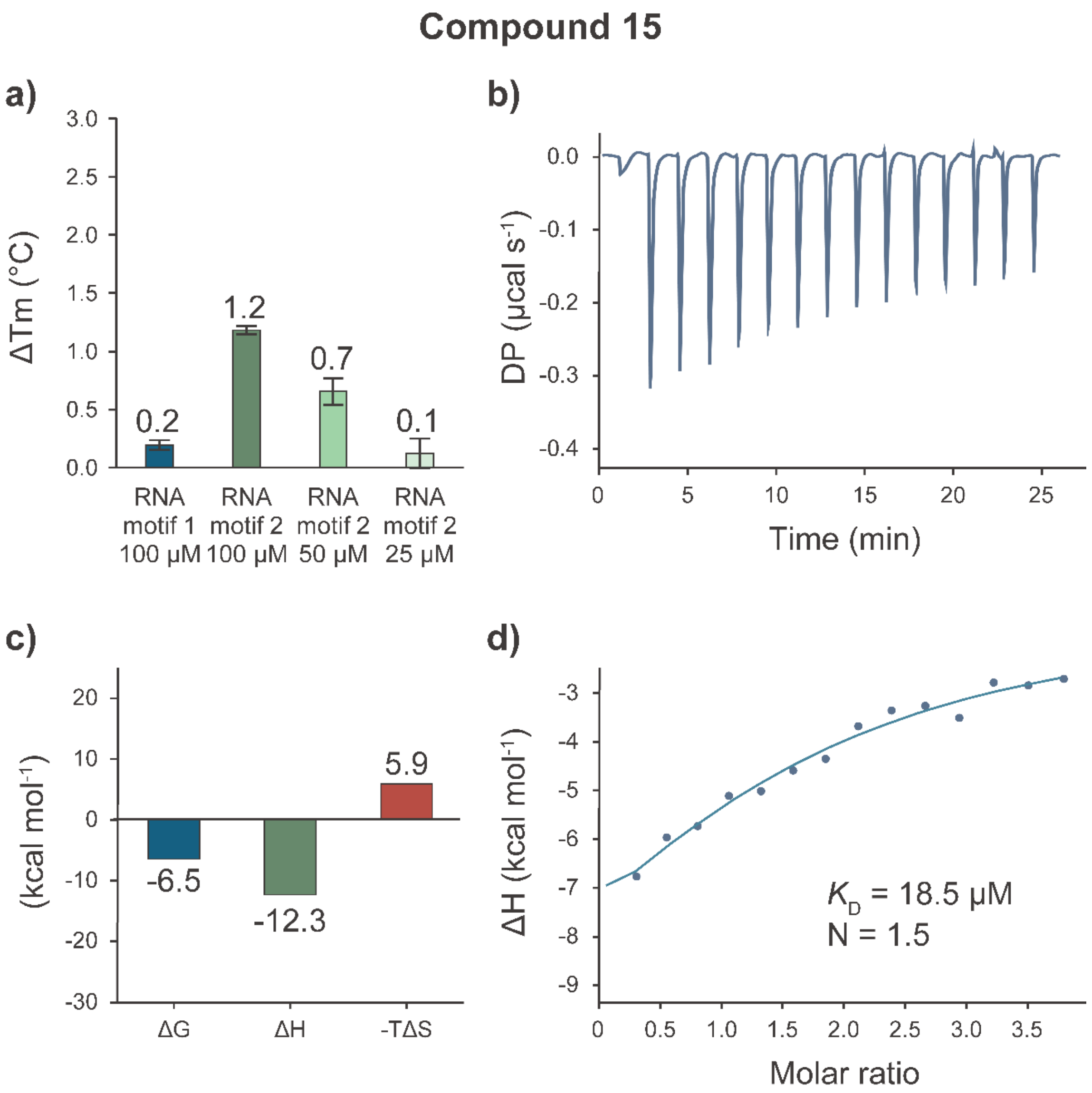

Figure S6. Biophysical characterization of compound **15** binding to RNA motifs. (**a**) qFRET dose–response analysis showing binding to RNA motif 1 (blue) and RNA motif 2 (green). (**b**) ITC thermogram from titration of compound **15** into RNA motif 2. (**c**) Thermodynamic parameters derived from ITC data: Δ*G* (blue), Δ*H* (green), and -*T*Δ*S* (red). (**d**) Binding isotherm with fitted curve (single set of sites model), showing ΔH versus molar ratio.

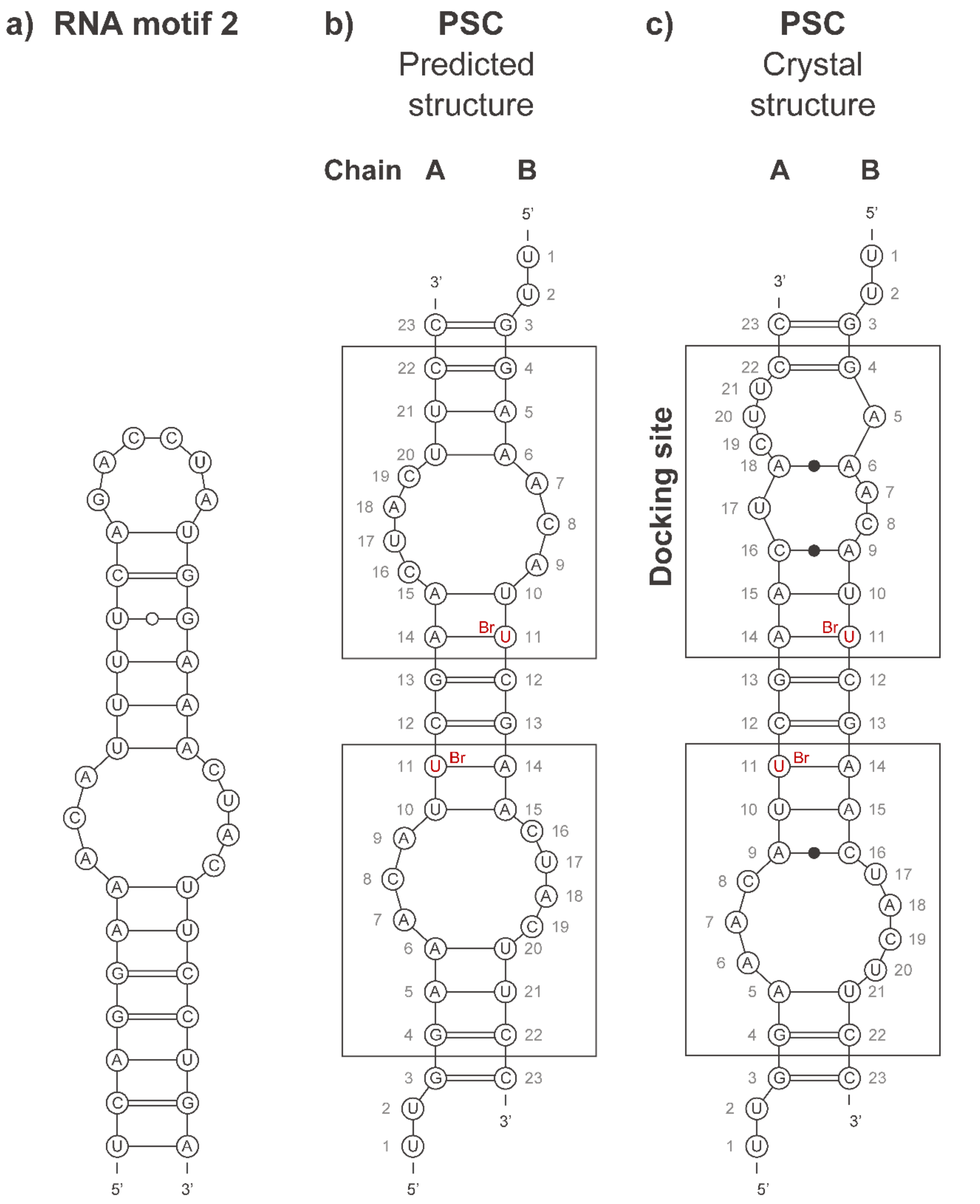

Figure S7. Secondary structures of RNA motif 2 and the pseudo-self-complementary (PSC) crystallization construct. (**a**) Predicted secondary structure of residues U187–A225 of p53 mRNA, referred to in this study as RNA motif 2. (**b**) Predicted secondary structure of PSC. (**c**) Secondary structure of PSC observed in the crystal structure. Bromouridines (BrU) are indicated in red.

Table S3. Crystal data, statistics of data collections, and structure refinements.

	9VSN
Crystal data	
Space group	$P2_12_12_1$
Unit cell (Å)	a = 25.5, b = 41.3, c = 64.6
Z[a]	1
Data collection	
Beamline	BL-17A of PF
Wavelength (Å)	1.0
Resolution (Å)	31.6-3.1
of the outer shell (Å)	3.2-3.1
Unique reflections	4463
Completeness (%)	98.5
in the outer shell (Å)	95.4
R_{anom}[b] (%)	10.1
in the outer shell (Å)	38.2
Redundancy	3.4
in the outer shell	3.2
Structure refinement	
Resolution range (Å)	31.6–3.1
Used reflections	4452
R-factor[c] (%)	22.3
R_{free}[d] (%)	26.4
RNA atoms	962
K^+ atoms	6
R.m.s.d. bond length (Å)	0.006
R.m.s.d. bond angles (°)	1.1

[a] Number of RNA duplex in the asymmetric unit. [b] $R_{anom}=100\times\Sigma_{hklj}|I_{hklj}(+)-I_{hklj}(-)|/\Sigma_{hklj}[I_{hklj}(+)+I_{hklj}(-)]$. [c] R-factor = $100\times\Sigma||F_o|-|F_c||/\Sigma|F_o|$, where $|F_o|$ and $|F_c|$ are optimally scaled observed and calculated structure factor amplitudes, respectively. [d] Calculated using a random set containing 10 % of observations.

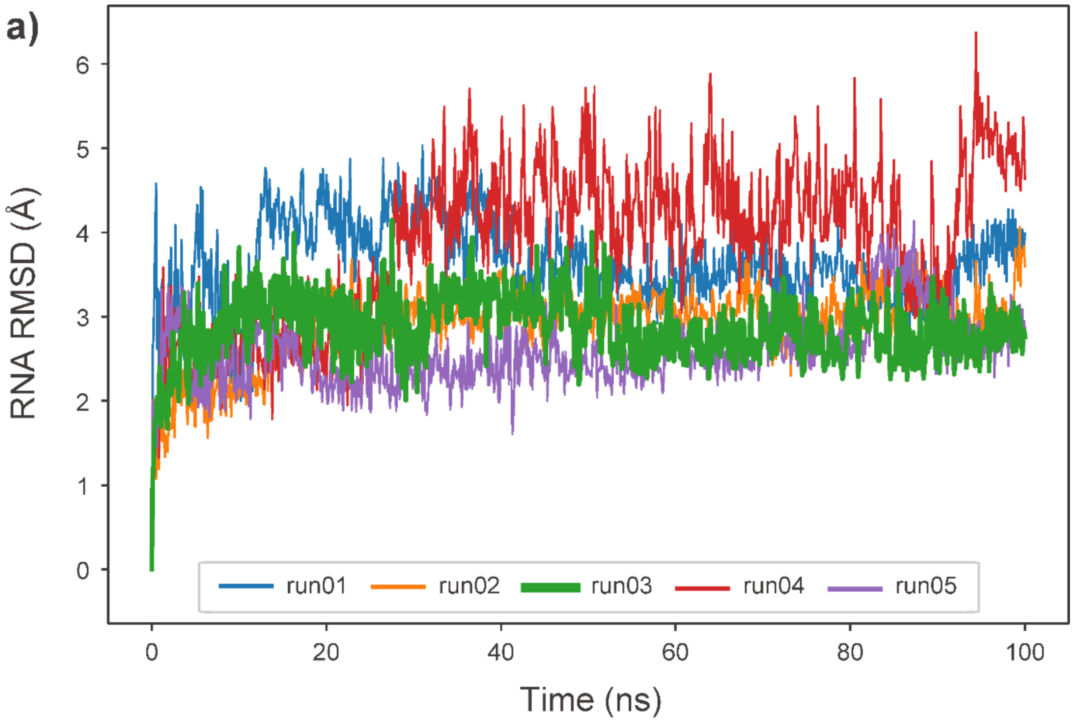

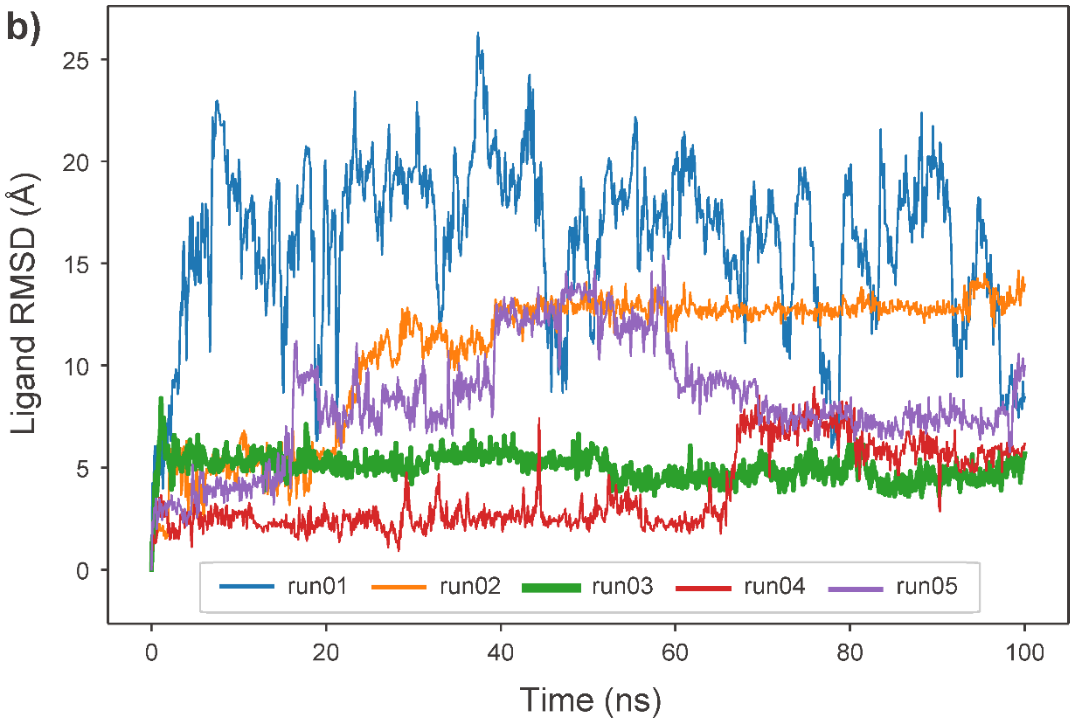

Figure S8. Time evolution of the root-mean-square deviation (RMSD) of the RNA (**a**) and the ligand (**b**) for five independent MD replicas. Although the absolute RMSD values are comparable among the replicas, one trajectory exhibits reduced temporal fluctuations and a

more stable RMSD profile over time, indicating a consistently maintained ligand-bound geometry.

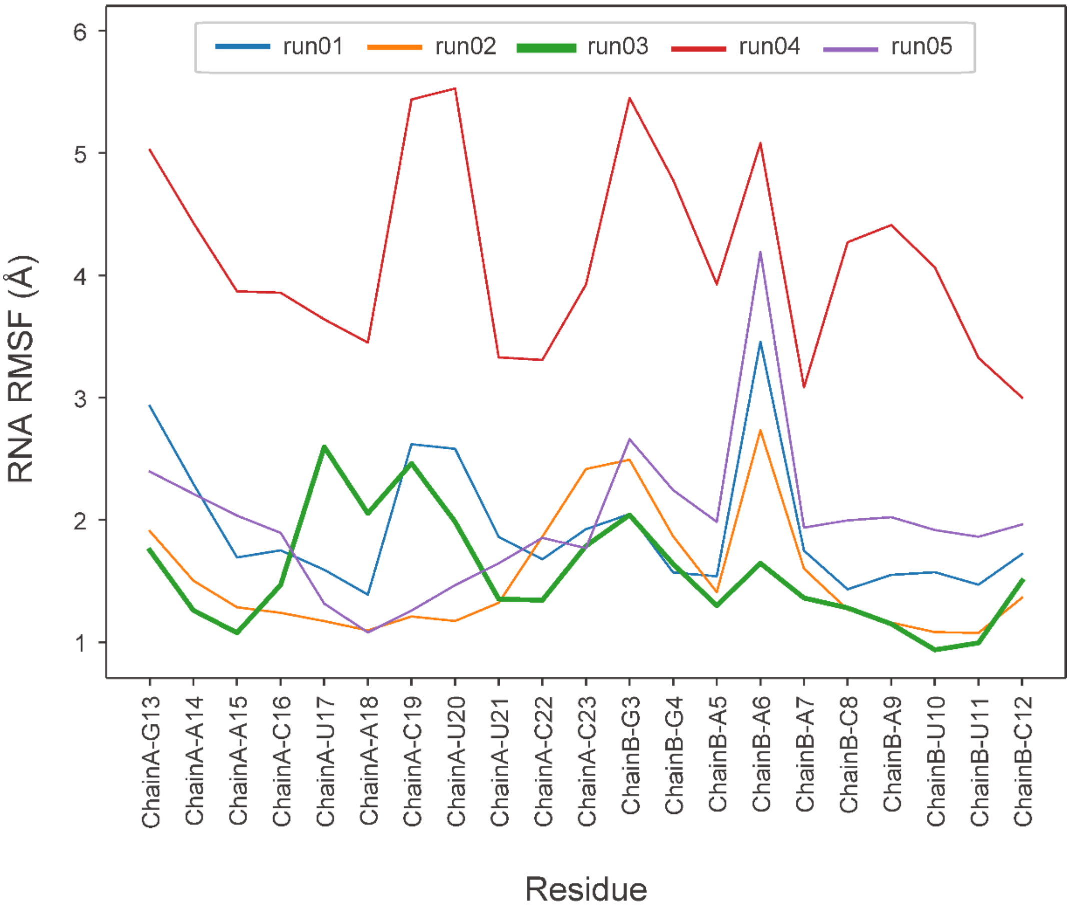

Figure S9. Per-residue root-mean-square fluctuation (RMSF) of the RNA calculated for each MD trajectory. While overall fluctuation profiles are similar across replicas, the selected trajectory shows notably reduced RMSF values for residues A5 and A7 in chain B, which form key interactions with the ligand, indicating localized stabilization upon ligand binding.

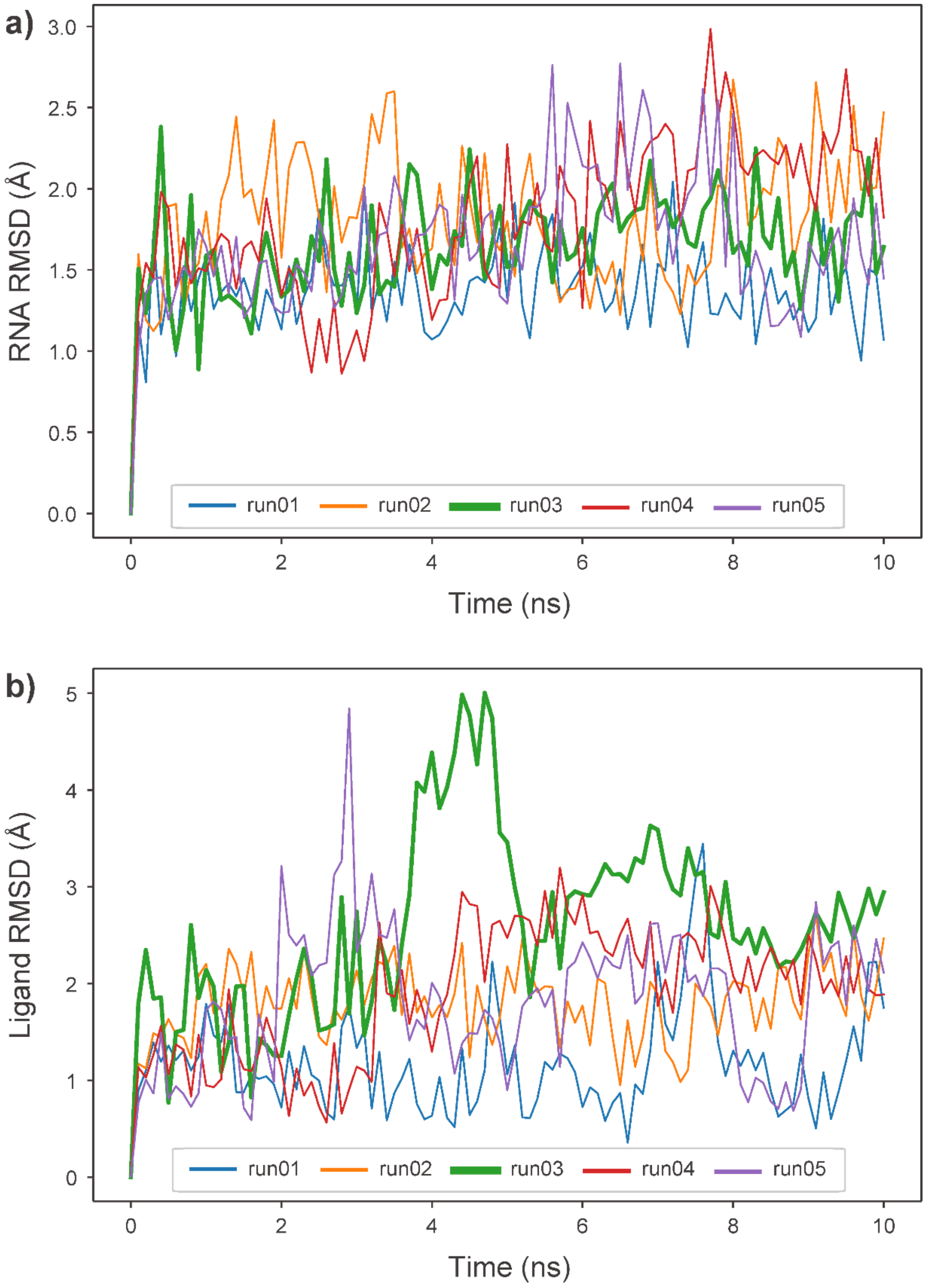

Figure S10. (**a**) RNA and (**b**) ligand RMSD profiles for five independent 10-ns MD simulations initiated from the representative ligand-bound structure. All replicas exhibit comparably low RMSD values with limited temporal fluctuations, indicating that the

extracted representative structure corresponds to a stable ligand-bound conformation under the simulation conditions.

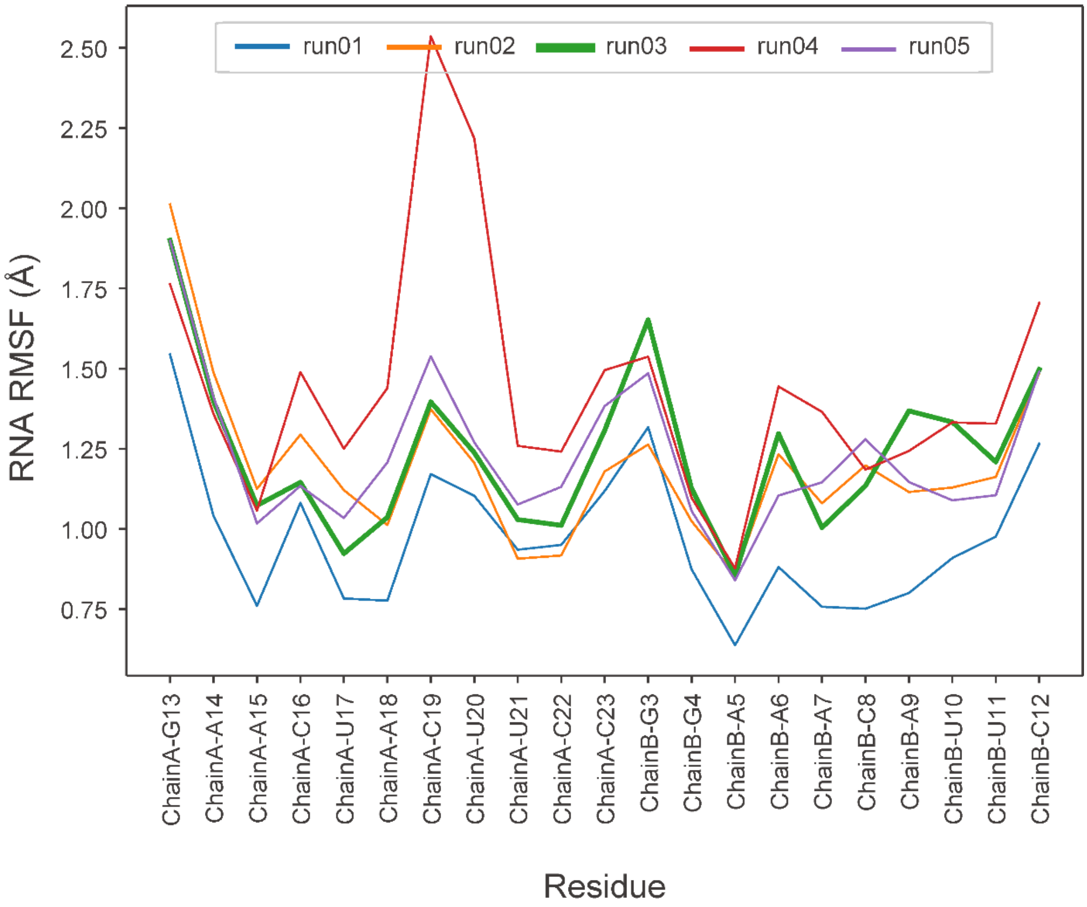

Figure S11. Per-residue RNA RMSF calculated from each of the five 10-ns MD replicas initiated from the representative structure. Overall fluctuations are low across the RNA, consistent with a stable complex. Notably, A5 and A7 in chain B, which form key contacts with the ligand, show particularly reduced RMSF values, indicating localized stabilization at the interaction site.

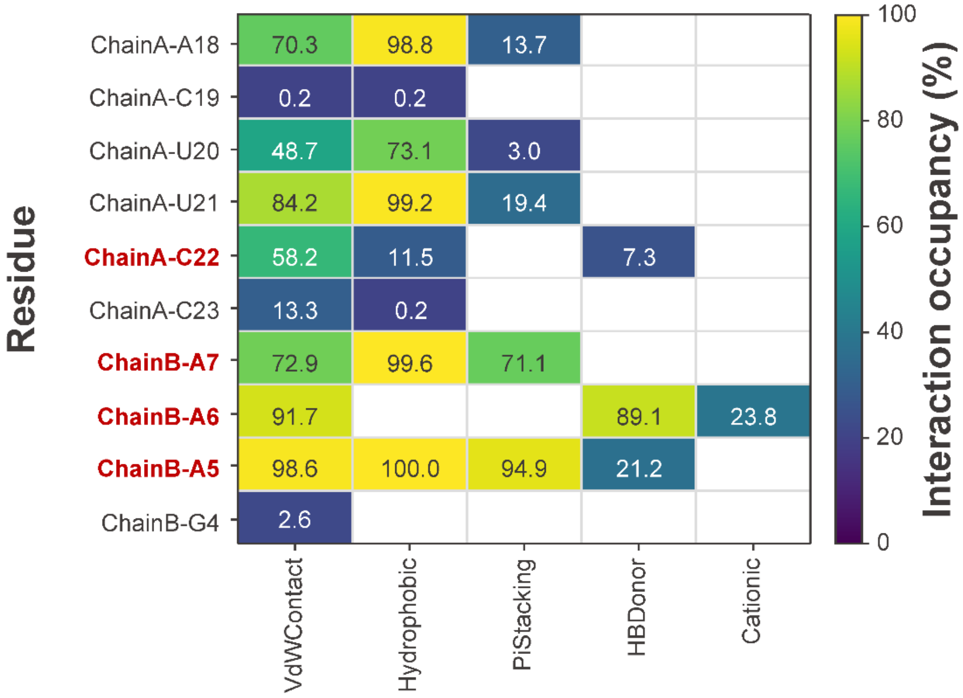

Figure S12. Heatmap of RNA–ligand interaction occupancies obtained from ProLIF analysis performed on the combined trajectories from five independent 10-ns MD replicas (run01–run05) initiated from the representative ligand-bound structure. Residues are ordered by sequence position across chains A and B, and interaction frequencies are reported as the percentage of frames in which each interaction was detected across the aggregated dataset. White cells indicate zero occupancy. Importantly, the overall interaction pattern is highly consistent with that observed in the original 100-ns simulation, including the dominance of aromatic stacking interactions involving chainB-A5 and chainB-A7, supporting the robustness of the identified binding mode.
